# Alternative Conceptual Approach to the Design of Bifunctional Catalysts: An Osmium Germylene System for the Dehydrogenation of Formic Acid

**DOI:** 10.1021/acs.inorgchem.1c02893

**Published:** 2021-10-16

**Authors:** María L. Buil, Javier A. Cabeza, Miguel A. Esteruelas, Susana Izquierdo, Carlos J. Laglera-Gándara, Antonio I. Nicasio, Enrique Oñate

**Affiliations:** † Departamento de Química Inorgánica, Instituto de Síntesis Química y Catálisis Homogénea (ISQCH), Centro de Innovación en Química Avanzada (ORFEO-CINQA), Universidad de Zaragoza-CSIC, 50009 Zaragoza, Spain; ‡ Departamento de Química Orgánica e Inorgánica, Centro de Innovación en Química Avanzada (ORFEO-CINQA), Universidad de Oviedo, 33071 Oviedo, Spain

## Abstract

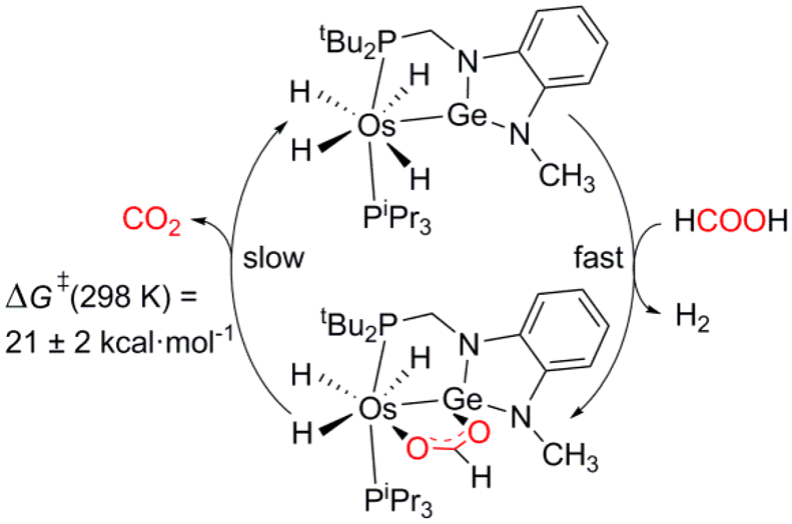

The reaction of the hexahydride OsH_6_(P^i^Pr_3_)_2_ with a P,Ge,P-germylene-diphosphine affords an osmium tetrahydride derivative bearing a Ge,P-chelate, which arises from the hydrogenolysis of a P–C(sp^3^) bond. This Os(IV)–Ge(II) compound is a pioneering example of a bifunctional catalyst based on the coordination of a σ-donor acid, which is active in the dehydrogenation of formic acid to H_2_ and CO_2_. The kinetics of the dehydrogenation, the characterization of the resting state of the catalysis, and DFT calculations point out that the hydrogen formation (the fast stage) exclusively occurs on the coordination sphere of the basic metal center, whereas both the metal center and the σ-donor Lewis acid cooperatively participate in the CO_2_ release (the rate-determining step). During the process, the formate group pivots around the germanium to approach its hydrogen atom to the osmium center, which allows its transfer to the metal and the CO_2_ release.

## Abstract 2

An alternative class of bifunctional catalysts can be assembled by coordination of σ-donor Lewis acids to platinum-group-metal basic fragments. In contrast to what happens with the previously reported bifunctional catalysts, this design allows enhancing the basicity of the base and the acidity of the acid. According to this, a bifunctional catalyst for the dehydrogenation of formic acid, based on an osmium(IV)-germylene cooperative system, has been prepared and the mechanism of the catalysis established.

## Introduction

**Chart 1:**
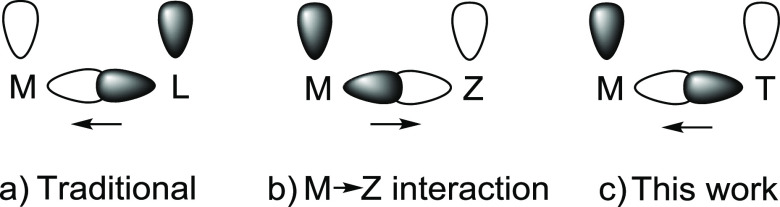
Bifunctional-Type Catalysts

The direct participation of both the metal center and a ligand of the catalyst during a transition-metal-catalyzed organic transformation is a successfully employed strategy in the current homogeneous organometallic catalysis. The metal–ligand cooperation is based on the distribution of roles among the main actors of the catalyst. In most of the cases, the metal acts as a Lewis acid whereas the ligand is a Lewis base (**a** in Chart 1).^1^ Such a principle is the support of most relevant catalysts of this class, including Shvo’s cyclopentadienone metal complexes,^2^ Fujita’s iridium pyridonate,^3^ Noyori-Ikariya’s ruthenium amide,^4^ or Milstein’s PNN-metal systems.^5^ Nevertheless, the design of bifunctional catalysts to operate with exchanged metal–ligand roles has awakening great interest in the last few years.^6^ Such catalysts are formed by a basic metal and an acidic σ-acceptor Z-type ligand where the bonding σ-orbital is empty (**b** in Chart 1).^7^ Unfortunately, the number of Z-type ligands is notably limited^8^ and as a consequence the reactions employing catalysts of this class are still very scarce. Metal Lewis bases mainly involve 3d metals, with iron,^9^ cobalt,^10^ and nickel^11^ in prominent positions, in addition to rhodium,^12^ palladium,^13^ platinum,^14^ and gold.^15^ The Lewis acid site of the ligands is boron^9,10a−10c,11b−11d,13a,15b,15d^ in the great majority of the cases and to a much lesser extent aluminum,^11a^^15a^ gallium,^11e^^11f^ indium,^12^ silicon,^13b^ and antimony.^7b,14,15c^ Catalytic reactions include oligomerization of primary silanes,^11a^ sila-Negishi couplings,^13b^ hydrosilylation of aldehydes,^10c^^11c^ olefin and alkyne hydrogenations,^9,11b,11d,11f^ CO_2_ hydrogenation to formate,^11e^ amine–borane dehydrogenation,^10a,10b^ cycloisomerization of propargylamides,^15a^ hydroaminations,^15c,15d^ C–H amination,^12^ enyne cyclization,^14,15b^ and dehalogenation of aryl chlorides.^13a^

The electron density flow of the metal–ligand interaction in both classes of bifunctional catalysts goes from the Lewis base to the Lewis acid, as usual. However, that works against the nature of both, since it decreases the basicity of the former and the acidity of the latter. We now address an alternative approach (**c** in Chart 1): the assembly of a bifunctional catalyst carrying such a flow in favor of both actors, enhancing the basicity of the base and the acidity of the acid. The challenge needed a basic center able of accepting electron density from an acidic center capable of donating it. We reasoned that such a situation would be possible by means of the use of a basic metal center, which should be in a high oxidation state, in contrast to those employed in catalysts involving a M→Z interaction. The high oxidation state should favor the acceptance of electron density from a σ-donor orbital and prevent significant back-bonding donation to an empty π-orbital. Metal fragments with these characteristics are the polyhydrides of the platinum-group metals.^16^ Among them, the osmium polyhydrides occupy a predominant position due to their proven ability to catalyze interesting organic synthesis reactions,^17^ in addition to the dehydrogenation of liquid organic hydrogen carriers^18^ and boranes.^19^ Promising acidic ligands capable of donating σ-electron density to a platinum-group metal in a high oxidation state are the heavier tetrylenes,^20^ heavier counterparts of Fisher-type carbenes. They prefer to situate the two free electrons in an orbital with marked s character, leaving a vacant orbital with high p character at an orthogonal plane. The metal–tetrylene bonding interaction is significantly weaker than the metal–carbene interaction, and its strength decreases on going down group 14. Thus, germylenes are particularly interesting due to the center position of germanium in the group, which grants a reasonable σ-donor ability and a low π-acceptor capacity to them.^21^ The latter is evidenced by the marked tendency of these ligands to undergo inter- and intramolecular addition of 2e^–^ donor groups.^22^

Formic acid is a polar molecule that is able of interacting with a polar M–L bond. Furthermore, because it is an attractive member of the family denoted as liquid organic hydrogen carriers, due to its low toxicity and flammability and reasonable gravimetric and volumetric hydrogen content (4.4 wt % and 53.4 g/L, respectively),^23^ its dehydrogenation to H_2_ and CO_2_ is of great interest. The reaction is catalyzed by both homogeneous and heterogeneous systems, including bifunctional catalysts based on the traditional acidic-metal/basic-ligand approach.^24^ The catalysis is divided into two main stages: H_2_ formation and CO_2_ formation.^25^ A mechanistic feature of the traditional bifunctional catalysts is the participation of both the metal and the ligand in the H_2_ formation stage, whereas the CO_2_ formation exclusively takes place on the metal coordination sphere.^26^ Thus, the dehydrogenation of formic acid might be an ideal reaction to be used as a proof of concept validation for our hypothesis and also to analyze the mechanistic differences between the traditional bifunctional catalysis and that now designed.

This paper reports the preparation of a bifunctional catalyst for the dehydrogenation of formic acid, based on an osmium(IV) germylene cooperative system, which works in a manner different from that of the traditional catalysts of this class and represents a new conceptual approach to the design of bifunctional catalysts.

## Results and Discussion

### Preparation of the Catalyst

In the search for a catalyst with a robust skeleton, which provides a strong base–acid interaction to the system, we introduced the germylene acid between two phosphine groups, with the initial idea of giving it the role of the central moiety of a neutral P,Ge,P-pincer ligand. With this aim, we chose 1,3-bis(di-*tert*-butylphosphanylmethyl)-1,3-dihydro-2λ^2^-benzo[*d*][1,3,2]diazagermole, because it had previously shown a notable versatility in platinum-group-metal chemistry.^27^ With this molecule in hand, we selected the d^2^ hexahydride OsH_6_(P^i^Pr_3_)_2_ (**1**) as the metal precursor, as it had proven to be efficient for the preparation of the tetrahydride OsH_4_{κ^3^-*P*,*O*,*P*-[xant(P^i^Pr_2_)_2_]} by the replacement of the triisopropylphosphines and a hydrogen molecule with the neutral P,O,P-pincer ligand 9,9-dimethyl-4,5-bis(diisopropylphosphino)xanthene.^17e^

**Scheme 1:**
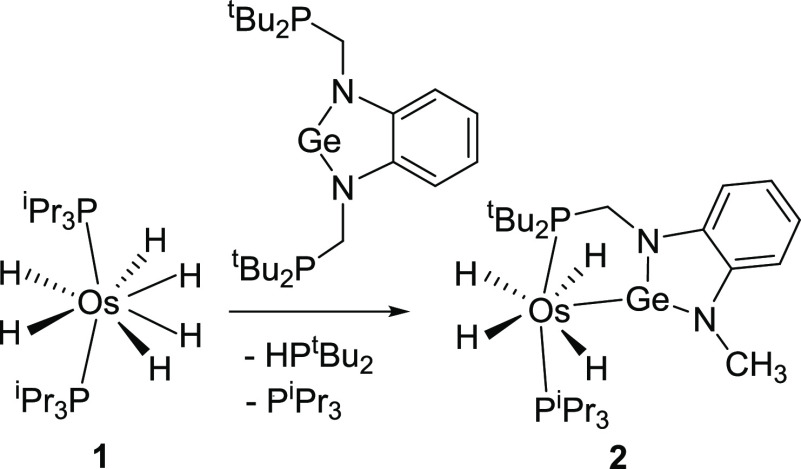
Formation of **2**

Treatment of toluene solutions of **1** with 1.1 equiv of the P,Ge,P-pincer, at 110 °C, for 18 h led to the coordination of the germylene moiety to the osmium center, as expected. However, the substitution of only one triisopropylphosphine ligand was observed, while the hydrogenolysis of the CH_2_–P^t^Bu_2_ bond of one of the pincer side arms took place. As a result, di-*tert*-butylphosphine and the tetrahydride **2**, bearing one of the initial triisopropylphosphines and a Ge,P-chelating germylene-phosphine ligand, were formed (Scheme 1). The elimination of di-*tert*-butylphosphine from the original P,Ge,P-pincer is notable. In spite of it being argued as the reason for the degradation of some hydrogenation catalysts^28^ and of being known for the rupture of P–C bonds of some quaternary phosphonium salts under catalytic conditions,^29^ the hydrogenolysis of P–C bonds is a slightly common reaction. It should be furthermore mentioned that, although complex **1** has shown a notable ability to activate σ-bonds,^30^ including C–C,^31^ C–N,^32^ and C–O^33^ among others, it had never participated in the rupture of a C–P bond.

**Figure 1:**
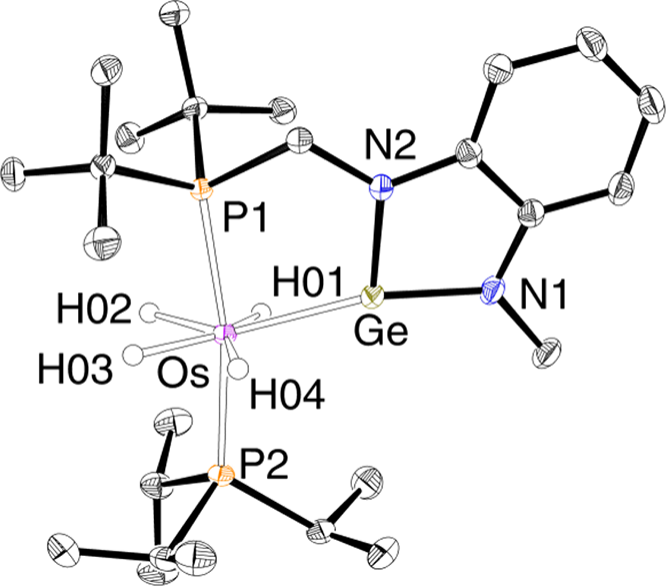
Molecular diagram of complex **2** (50% probability ellipsoids). Hydrogen atoms (except hydride ligands) are omitted for clarity. Selected bond lengths (Å) and angles (deg): Os–Ge = 2.3593(3), P(1)–Os–P(2) = 169.88(2), P(1)–Os–Ge = 81.038(16), P(2)–Os–Ge = 108.668(17), N(2)–Ge–N(1) = 86.49(9), N(1)–Ge–Os = 161.01(7), N(2)–Ge–Os = 110.75(6).

Complex **2** was isolated as a pale yellow solid in 48% yield and characterized by X-ray diffraction analysis. Figure 1 gives a view of the structure. The coordination polyhedron around the osmium atom can be rationalized as a pentagonal bipyramid with the phosphine P atoms at the apical positions (P(1)–Os–P(2) = 169.88(2)°), whereas the hydride ligands and the germylene group define the base of the polyhedron. The Os–Ge distance, 2.3593(3) Å, compares well with those reported for other osmium germylene derivatives.^34^ A DFT-optimized structure confirmed the classical character of the hydride ligands, since it reveals a separation between them of longer than 1.834 Å. In toluene, these ligands are involved in thermally activated position exchange processes. Thus, the ^1^H NMR spectrum at room temperature shows only one high-field resonance for the two groups of inequivalent hydrides, which appears at −10.45 ppm as a doublet of doublets, with both H–P coupling constants of 12.4 Hz. Lowering the temperature of the sample produces a broadening of the resonance. However, decoalescence is not reached even at 183 K. In the same temperature range, the ^31^P{^1^H} NMR spectrum contains two doublets at 99.8 and 55.5 ppm. In agreement with the mutually *trans* disposition of the phosphorus nuclei, the P–P coupling constant is 224.5 Hz.

Complex **2** is not exactly the compound initially designed by us but is very similar. The main difference is the lack of a link between the germylene group and the monodentate phosphine. Because complex **2** still fulfills the planned features regarding the Os–Ge interaction, we decided to pursue the initial program.

### Reactions of **2** with Benzoic and Acetic Acids

**Scheme 2:**
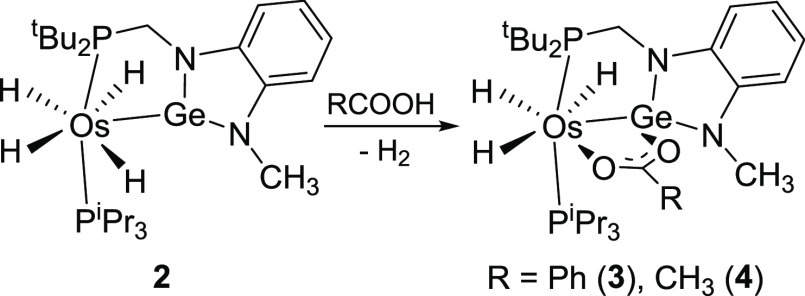
Reactions of **2** with Benzoic and Acetic Acids

The OsH_4_ moiety is certainly the basic part of **2**, whereas the acid center is located at the germanium atom. In agreement with this, complex **2** reacted with carboxylic acids in toluene at room temperature to give the trihydrides **3** and **4** (Scheme 2). These compounds result from the protonation of the basic osmium moiety and the neutralization of the Lewis acidity of the germylene by an arm of the corresponding carboxylate anion. The protonation generates a dihydrogen ligand that is released, occupying the generated coordination vacancy of the other arm of the carboxylate groups.

**Figure 2:**
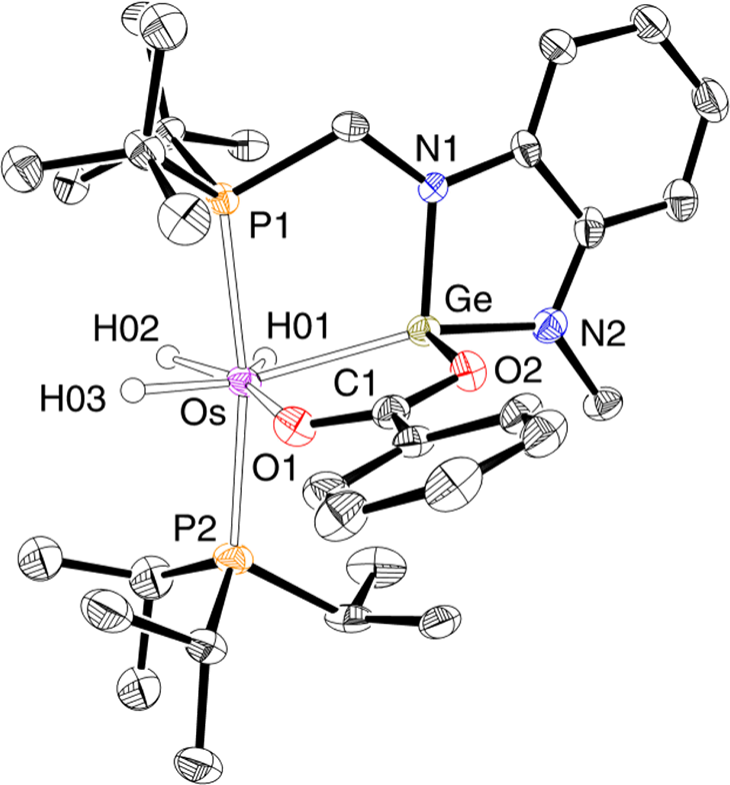
Molecular diagram of complex **3** (50% probability ellipsoids). Hydrogen atoms (except hydride ligands) are omitted for clarity. Selected bond lengths (Å) and angles (deg): Os–Ge = 2.4204(7), Os–O(1) = 2.154(5), Ge–O(2) = 2.042(4), O(1)–C(1) = 1.267(8), O(2)–C(1) = 1.272(8), P(1)–Os–P(2) = 170.07(6), P(1)–Os–Ge = 81.22(4), P(2)–Os–Ge = 108.45(5), P(1)–Os–O(1) = 97.11(13), Ge–Os–O(1) = 79.87(12), N(2)–Ge–N(1) = 86.7(2), N(2)–Ge–O(2) = 100.3(2), N(1)–Ge–O(2) = 98.9(2), N(1)–Ge–Os = 108.98(15), N(2)–Ge–Os = 155.50(17), O(2)–Ge–Os = 95.75(12).

Complexes **3** and **4** were isolated as pale yellow solids in 56% and 48% yields, respectively. Their formation was confirmed by a structural X-ray diffraction analysis of **3** (Figure 2). The addition of the anion to the germanium atom gives rise to a P,Ge,O-tridentate ligand, which coordinates in a *fac* fashion to the metal center, with bite angles P(1)–Os–Ge, P(1)–Os–O(1), and Ge–Os–O(1) of 81.22(4), 97.11(13), and 79.87(12)°. The resulting coordination polyhedron around the osmium atom is the typical pentagonal-bipyramidal characteristic of a osmium(IV) polyhydride species with the phosphorus atoms in apical positions (P(1)–Os–P(2) = 170.07(6)°), whereas the hydride ligands and the germanium and oxygen atoms of the tridentate group lie at the base. The Os–Ge bond length of 2.4204(7) Å is about 0.06 Å longer than in **2**.The DFT-optimized structure revealed that the OsH_3_ unit forms a hydride-compressed dihydride system with H(01) and H(02) separated by 1.540 Å and H(02) and H(03) by 1.752 Å. As is usual in this class of polyhydrides, the hydride ligands are involved in thermally activated positional exchange processes in solution. Thus, the ^1^H NMR spectra of both species, in toluene-*d*_8_, at room temperature display a broad resonance at about −11.6 ppm for the inequivalent hydride ligands. In contrast to **2**, this resonance decoalesces between 243 and 233 K, to afford a broad triplet around at −4 ppm and an AB spin system close to −15.5 ppm. The H–H coupling constant values of the latter, >124 Hz, suggest that the hydrogen nuclei of the compressed dihydride undergo quantum exchange coupling^16,32b,35^ in addition to the thermally activated positional exchange process. In agreement with **2**, the ^31^P{^1^H} NMR spectra show two doublets at about 84 and 48 ppm with P–P coupling constants of around 236 Hz.

### Reaction of **2** with Fomic Acid

**Scheme 3:**
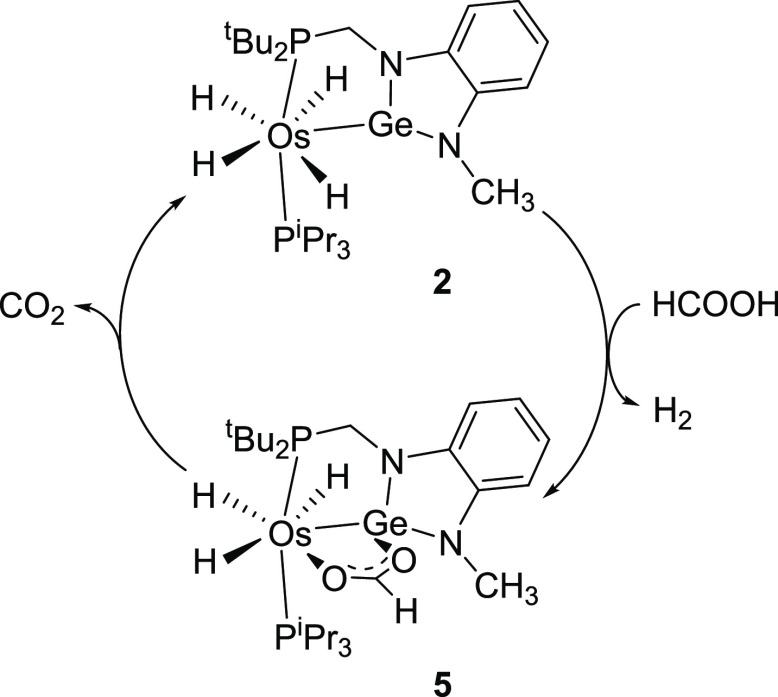
Stoichiometric Cycle for the Dehydrogenation of Formic Acid to H_2_ and CO_2_ Promoted by **2**

Once it was known that complex **2** activates the O–H bond of usual carboxylic acids, such as benzoic and acetic acids, and established the nature of the resulting compounds, we investigated the behavior of formic acid. The addition of 1.0 equiv of the latter to toluene solutions of the tetrahydride, at room temperature, rapidly produced the release of 1.0 equiv of H_2_ and the quantitative formation of **5**, the formate counterpart of **3** and **4**. Complex **5** was fully characterized by ^1^H, ^31^P{^1^H}, and ^13^C{^1^H} NMR spectroscopy (see the Supporting Information). In agreement with **3** and **4**, the ^1^H spectrum of **5** contains a broad resonance centered at −11.73 ppm, which is split into a broad signal at −4.16 ppm and an AB spin system at −15.66 ppm (^2^*J*_H–H_ > 153.0 Hz) at temperatures lower than 233 K, whereas the ^31^P{^1^H} spectrum displays two doublets at 84.1 and 48.3 ppm with a P–P coupling constant of 234.6 Hz. In solution, complex **5** is unstable. It releases CO_2_ to regenerate **2** (Scheme 3).

**Figure 3:**
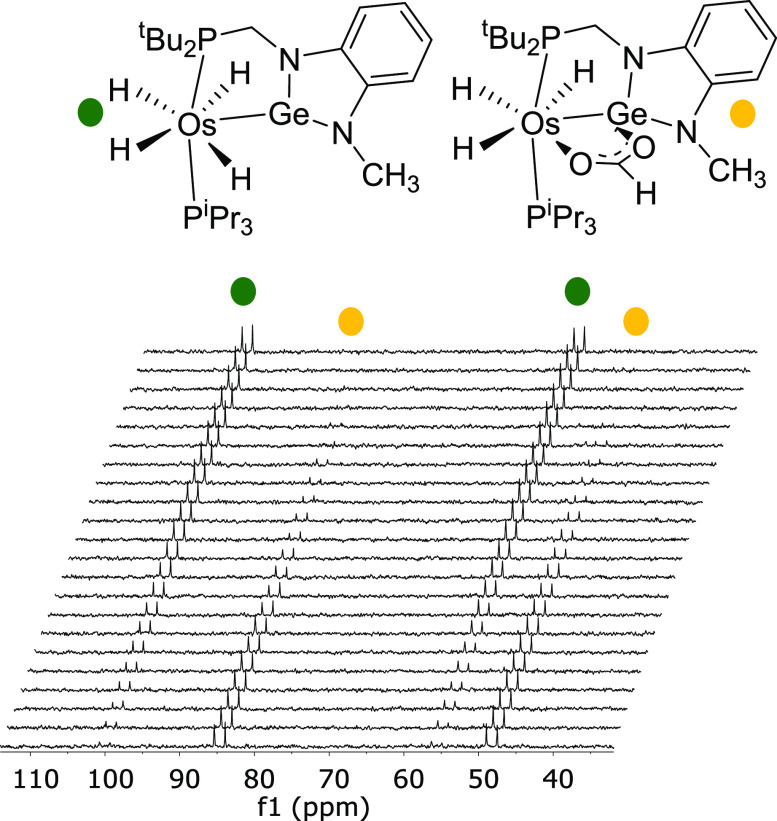
^31^P{^1^H} spectra (161.98 MHz, in toluene-*d*_8_) for the decarboxylation of **5** at 323 K.

**Figure 4:**
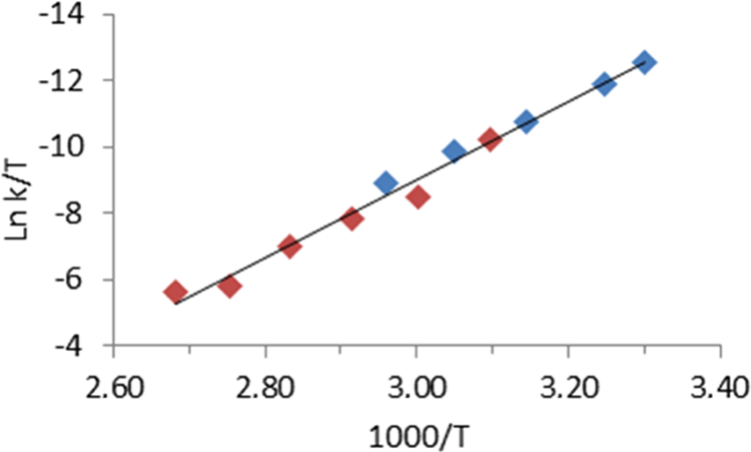
Eyring plot (blue: transformation of **5** into **2**; red, catalytic dehydrogenation of formic acid).

The formation of **5** and its decomposition constitutes a cycle for the stoichiometric dehydrogenation of formic acid into H_2_ and CO_2_, which is kinetically controlled by the CO_2_ release, as illustrated Scheme 3. To gain information about the activation parameters of the process, we followed the transformation of **5** into **2** by ^31^P{^1^H} NMR spectroscopy, between 303 and 338 K, as a function of time. Figure 3 shows the spectra of the reaction at 323 K. The consumption of **5** is an exponential function of time, which fits a first-order process, according to the expression
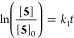
1where [**5**]_0_ is the initial concentration of **5** and [**5**] is the concentration at time *t*. Table 1 gathers the values obtained for *k*_1_ in the studied temperature range. The activation parameters calculated through an Eyring analysis (Figure 4) are Δ*H*_1_^⧧^ = 21 ± 2 kcal mol^–1^ and Δ*S*_1_^⧧^ = −3 ± 5 cal K^–1^ mol^–1^. These values yield an activation energy Δ*G*_1_^⧧^ at 298 K of 22 ± 3 kcal mol^–1^.

**Table 1:** Rate Constants for the Transformation of **5** into **2**

temp (K)	rate constant *k*_st_ (min^–1^)
303	0.001
313	0.002
318	0.007
328	0.017
338	0.045

### Dehydrogenation of Formic Acid Catalyzed by **2**: Kinetics and Mechanism

As expected from Scheme 3, complex **2** catalyzes the dehydrogenation of formic acid to H_2_ and CO_2_. The catalysis was carried out in toluene, in a closed reactor under constant-volume conditions, between 333 and 373 K. To see whether the cycle of Scheme 3 is furthermore catalytic, the kinetics of the catalysis was also studied under pseudo-first-order conditions. The partial pressure of the generated hydrogen (*P*_H_2__; atm) was determined according to eq 2, where *P*_T_ and *P*_CO_2__ are the total pressure and CO_2_ partial pressure, respectively.
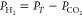
2

**Figure 5:**
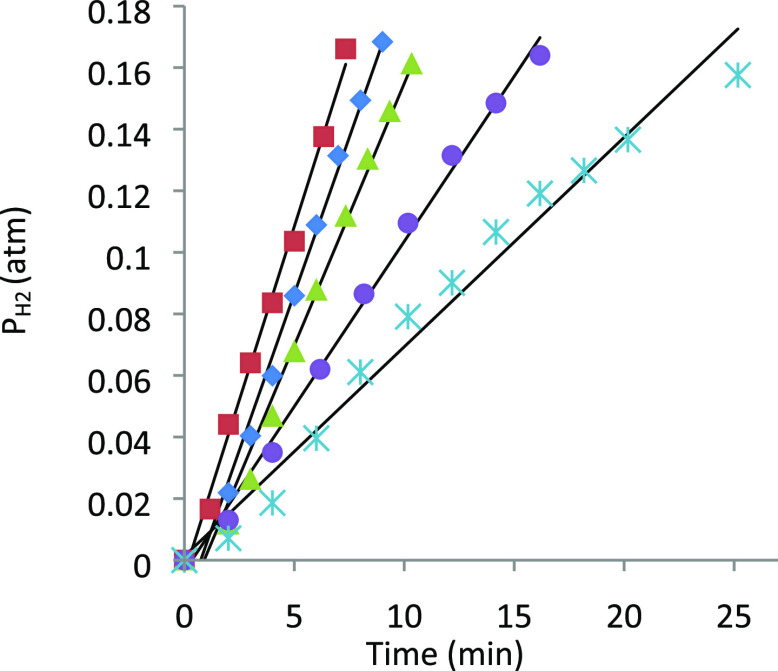
Plot of *P*_H_2__ vs time (*T* = 373 K, [HCOOH]_0_ = 0.26 M, 10^2^[**2**] = 0.26 M (pale blue asterisks), 0.53 M (purple circles), 0.79 M (green triangles), 1.06 M (blue diamonds), 1.32 M (red squares)).

Table 2 collects initial rates obtained from the gas evolution experiments by graphing the expression shown in eq 3, where *V* is the volume of the reactor (L), *R* is the molar gas constant, *T* is the temperature (K), and *V*_sol_ is the volume of the catalytic solution (L). Figure 5 exemplifies the reactions completed at 373 K, with a concentration of formic acid of 0.26 M.
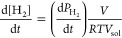
3

**Table 2:** Kinetic Data for the Dehydrogenation of Formic Acid Catalyzed by **2**

*T* (K)	[cat.] (10^2^ M)	[HCOOH] (M)	d[H_2_]/d*t* (10^2^ M min^–1^)	*k* (min^–1^)
373	0.26	0.26	0.241	0.912
373	0.53	0.26	0.589	1.110
373	0.79	0.26	0.830	1.045
373	1.06	0.26	0.895	0.845
373	1.32	0.26	1.277	0.965
373	0.53	0.40	0.589	1.112
373	0.53	0.53	0.465	0.877
373	0.53	0.66	0.459	0.866
373	0.53	0.79	0.477	0.899
363	0.53	0.26	0.306	0.577
353	0.53	0.26	0.159	0.300
343	0.53	0.26	0.100	0.189
333	0.53	0.26	0.088	0.167

A general rate law for the dehydrogenation of formic acid catalyzed by **2** is given by eq 4:
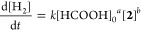
4

**Figure 6:**
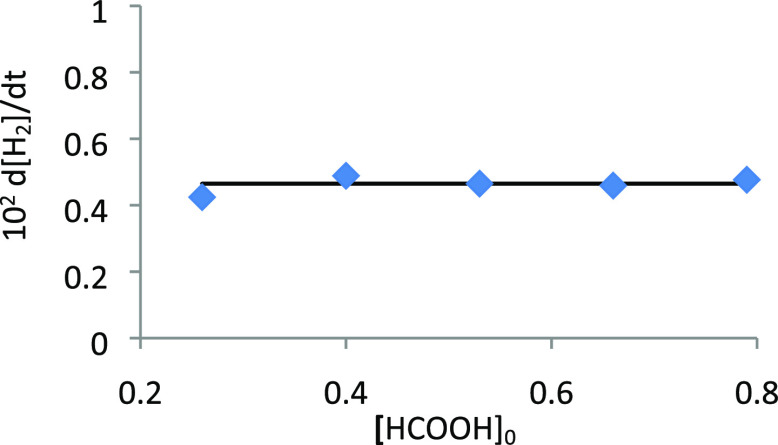
Plot of d[H_2_]/d*t* vs [HCOOH]_0_ (*T* = 373 K, [**2**] = 0.53 × 10^–2^ M).

**Figure 7:**
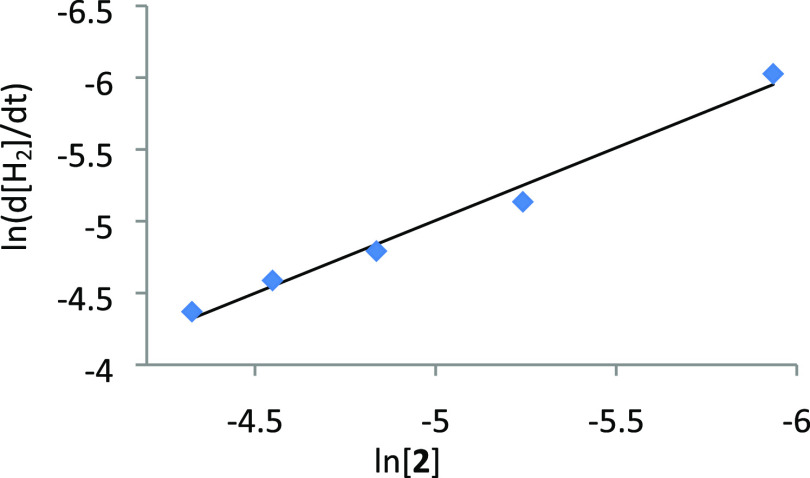
Plot of ln(d[H_2_]/d*t*) vs ln [**2**] (*T* = 373 K, [HCOOH]_0_ = 0.26 M).

The rate dependence on the formic acid concentration was studied at 373 K, for a concentration of **2** of 5.3 × 10^–3^ M, with variable initial concentrations of carboxylic acid ([HCOOH]_0_) between 0.26 and 0.79 M. Under these conditions, the dehydrogenation rate is independent of [HCOOH]_0_ (Figure 6), indicating that *a* = 0 in eq 4. The catalyst dependence was also investigated at 373 K, for [HCOOH]_0_ = 0.26 M, increasing the concentration of **2** from 2.6 × 10^–3^ to 1.32 × 10^–2^ M. In contrast to Figure 6, the plot of ln(d[H_2_]/d*t*) vs ln[**2**] gives a straight line of slope 1.01 (Figure 7), in accordance with a first-order dependence: i.e., *b* = 1 in eq 4. Therefore, the rate law for the dehydrogenation of formic acid catalyzed by **2** is
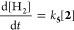
5

**Figure 8:**
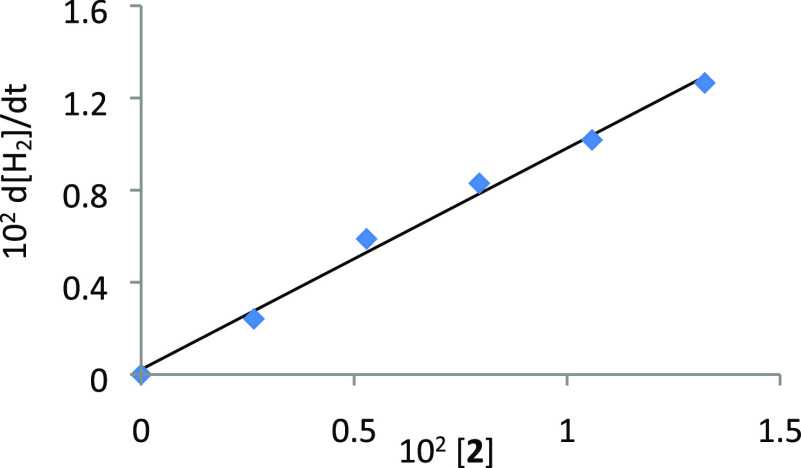
Plot of d[H_2_]/d*t* vs [**2**] (*T* = 373 K, [HCOOH]_0_ = 0.26 M).

A plot of d[H_2_]/d*t* vs [**2**] (Figure 8) yields for *k*_**5**_ a value of 0.96 ± 0.10 min^–1^ at 298 K. The corresponding Eyring analysis (Figure 4, red points) affords values of Δ*H*_5_^⧧^ = 19 ± 1 kcal mol^–1^, Δ*S*_5_^⧧^ = −7 ± 3 cal K^–1^ mol^–1^, and Δ*G*_5_^⧧^ = 21 ± 2 kcal mol^–1^ at 298 K. To our delight, they compare well with those obtained for the stoichiometric release of CO_2_ from **5**. As it should be, since both Eyring analyses fit the same straight line, which leads to common activation parameters for the catalysis and the stoichiometric release of CO_2_ from **5** of Δ*H*^⧧^ = 23 ± 1 kcal mol^–1^, Δ*S*^⧧^ = 4 ± 3 cal K^–1^ mol^–1^, and Δ*G*^⧧^ = 21 ± 2 kcal mol^–1^ at 298 K. Figure 4 gives overwhelming evidence supporting the catalytic nature of the cycle shown in Scheme 3, which was furthermore reinforced by the ^31^P{^1^H} NMR spectra of catalytic solutions quenched at half of the dehydrogenation. They reveal that complex **5** is the only osmium species present in a detectable concentration while formic acid is not completely consumed. The value of Δ*G*^⧧^ at 298 K lies in the central part of the range previously reported for this parameter in the dehydrogenation of formic acid catalyzed by other homogeneous systems (17–26 kcal mol^–1^).^18b,26c,36^

**Figure 9:**
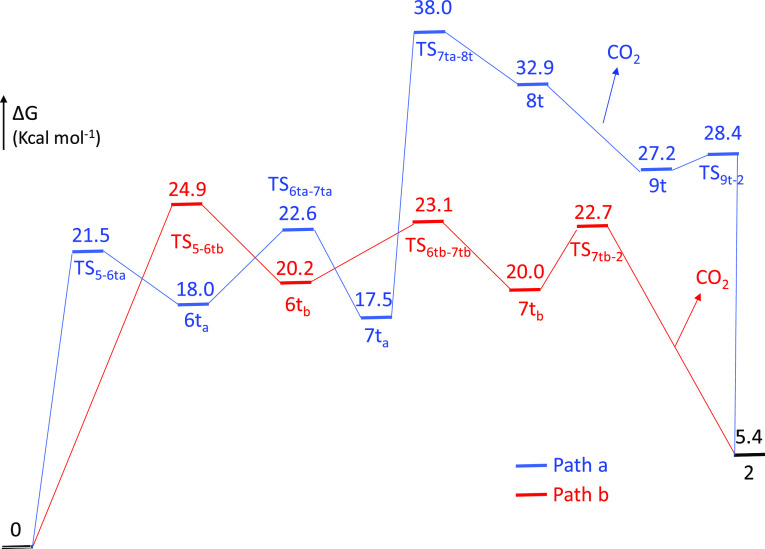
Energy profiles for the transformation of **5** into **2**.

**Scheme 4:**
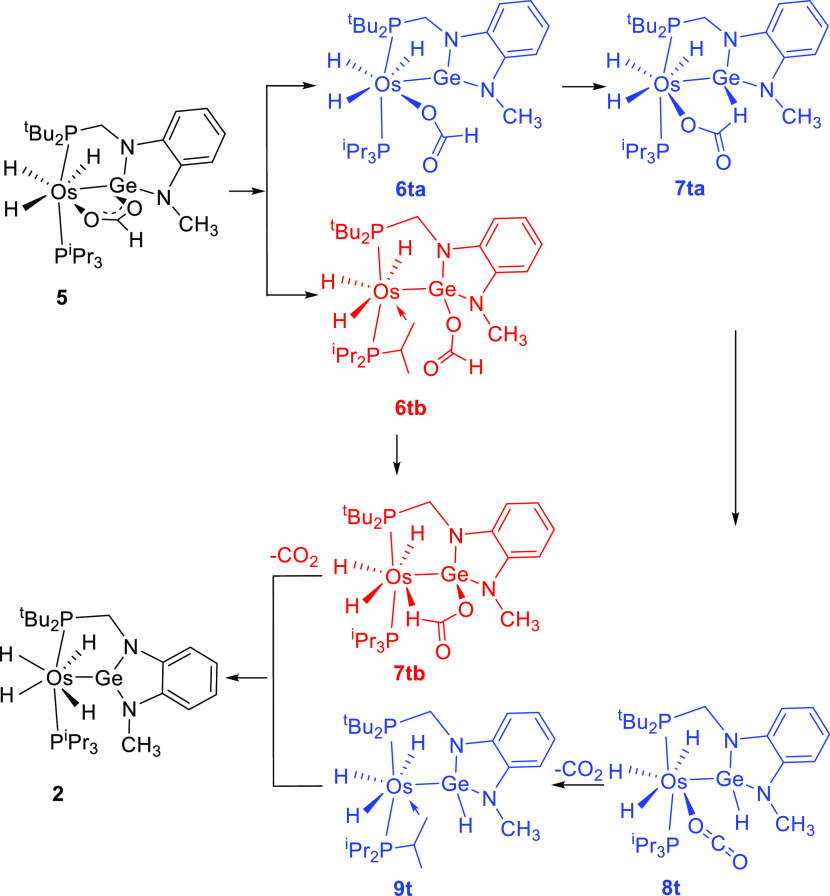
DFT-Calculated Intermediates for Paths a (Blue) and b (Red)

The dehydrogenation of formic acid catalyzed by **2** shows significant differences with regard to those promoted by traditional bifunctional catalysts, from a mechanistic point of view. In contrast to the traditional systems, the hydrogen formation (the fast stage) exclusively occurs on the coordination sphere of the basic metal center, whereas the CO_2_ release (the catalytic rate-determining step) is a cooperative process between the metal and the σ-donor Lewis acid. To gain information about the intimate details of the cooperation, we carried out DFT calculations (wB97XD/SDD/cc-pVDZ) with regard to the CO_2_ formation. The Δ*G* values were calculated in toluene at 298.15 K and 1 atm. Figure 9 shows the calculated profiles, whereas Scheme 4 collects the optimized intermediates.

**Chart 2:**
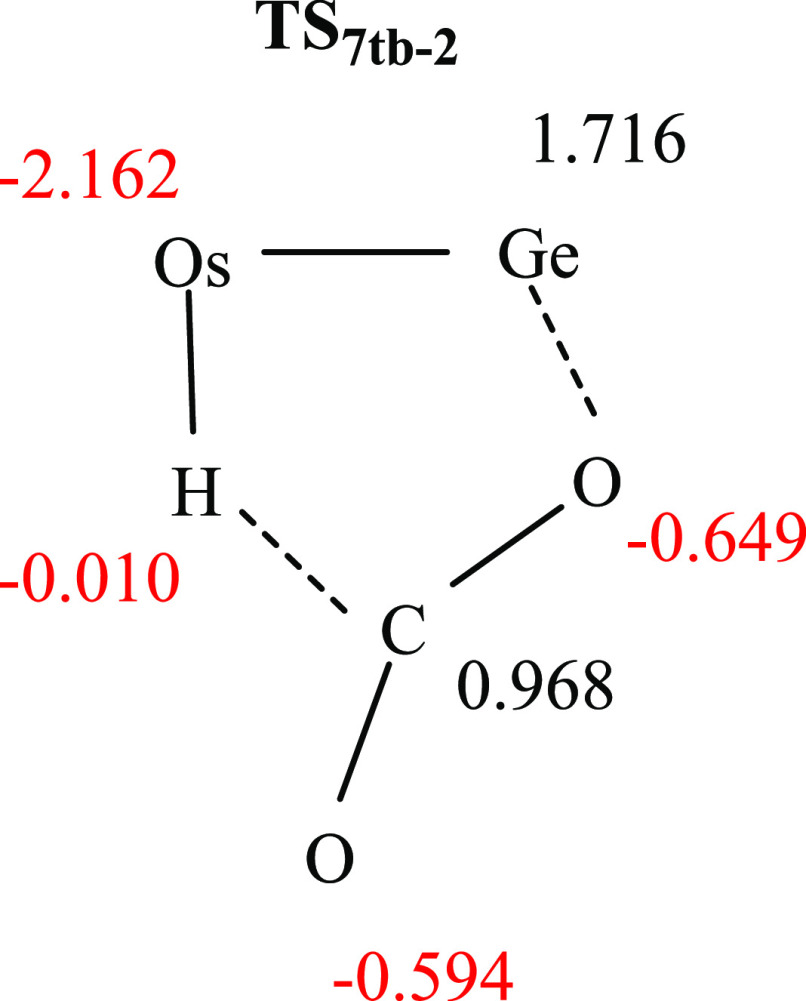
NBO Charges at **TS**_**7tb-2**_

The stoichiometric decomposition of **5** into **2** and CO_2_ is endergonic by 5.4 kcal mol^–1^, where the gas elimination acts as a driving force of the reaction. Under catalytic conditions, the absorbed energy is compensated by the exothermic hydrogen formation. The process can be started in two alternative ways (paths a and b), which involve the rupture of the Ge–O bond to form intermediate **6t**_**a**_ (path a) or the rupture of the Os–O bond to give intermediate **6t**_**b**_ (path b)_._ According to path a, once intermediate **6t**_**a**_ is formed, the monodentate formate ligand pivots around the Os–O bond to situate its hydrogen on the Ge atom. In this context, it should be noted that a β-hydrogen elimination of the formate hydrogen atom is not possible, due to the saturated character of the metal center. The resulting species **7t**_**a**_ evolves to **8t** by means of the rupture of the formate C–H bond. The subsequent release from the coordinated CO_2_ molecule leads to the germyl intermediate **9t**, which undergoes α-hydrogen elimination to regenerate **2**. This route must overcome an activation energy of 38.0 kcal mol^–1^, which is out of the range from the Δ*G*^⧧^ values experimentally obtained. Thus, path a must be rejected as a reasonable proposal. The unsaturated intermediate **6t**_**b**_, formed according to path b, saturates the metal coordination sphere by means of an agostic interaction with a methyl C–H bond of the triisopropylphosphine. Its formation takes place with an activation energy of 24.9 kcal mol^–1^. This value is the highest of the profile associated with path b and compares well with the experimental value of Δ*G*^⧧^, supporting path b as the only one possible. The formate group, which is now κ^1^-coordinated to the Ge atom, pivots around the latter to approach its hydrogen atom to the osmium center. As a result, the formate hydrogen atom displaces the methyl C–H bond from the osmium to afford **7t**_**b**_, which finally loses CO_2_ to yield **2**. The NBO charges at **TS**_**7tb-2**_ strongly support the basic character of the metal center and the acidic nature of the Ge atom (Chart 2).

CO_2_ formation usually occurs by β-hydrogen elimination on unsaturated M{κ^1^-OC(O)H} intermediates^37^ or hydrogen abstraction on saturated species of the same type. In the second case, the metal center of the catalyst slips from the coordinated oxygen into the hydrogen atom before releasing the CO_2_ molecule.^18a,36b,38^ The movement described herein is an alternative manner of implementing the CO_2_ release.

## Concluding Remarks

An alternative class of bifunctional catalysts can be assembled by coordination of σ-donor Lewis acids to platinum-group-metal basic fragments. In contrast to what happens with the previously reported bifunctional catalysts, this design allows enhancing the basicity of the base and the acidity of the acid. As a proof of this, we have prepared a bifunctional catalyst, for the dehydrogenation of formic acid to H_2_ and CO_2_, coordinating a bidentate phosphine-tetrylene ligand to an osmium(IV) tetrahydride metal fragment. Furthermore, we have discovered during the preparation of this complex that polyhydrides with a high number of hydride ligands, such as the osmium hexahydride OsH_6_(P^i^Pr_3_)_2_, are able to perform the hydrogenolysis of P–C(sp^3^) bonds.

The kinetics of the formic acid dehydrogenation, the characterization of the resting state of the catalysis along with the establishment of the activation parameters of its evolution, and DFT calculations regarding the rate-determining step prove that the prepared Os(IV)–Ge(II) catalyst works in a manner different from that of the traditional bifunctional catalysts, based on an acid metal fragment and a cooperative basic ligand. In contrast to the traditional systems, the hydrogen formation (the fast stage) exclusively occurs on the coordination sphere of the basic metal center, whereas the CO_2_ release (the rate-determining step) is a cooperative processes involving the metal and the σ-donor Lewis acid. The process taking place during the cooperation is also an alternative manner of implementing the CO_2_ release.

## Experimental Section

### General Information

General experimental details, X-ray analysis, spectroscopic and general instrumental techniques, and computational information are given in the Supporting Information. Chemical shifts are expressed in ppm, whereas the coupling constants *J* and *N* (*N* = *J*_H–P_ + *J*_H–P′_ for ^1^H and *N* = *J*_C–P_ + *J*_C–P′_ for ^13^C{^1^H)) are given in Hz. The complex OsH_6_(P^*i*^Pr_3_)_2_^39^ and 1,3-bis(di-*tert*-butylphosphanylmethyl)-1,3-dihydro-2λ^2^-benzo[*d*][1,3,2]diazagermole^27a^ were prepared as previously described.

### Preparation of **2**

The complex OsH_6_(P^*i*^Pr_3_)_2_ (332 mg, 0.643 mmol) and 1,3-bis((di-*tert*-butylphosphanyl)methyl)-1,3-dihydro-2λ^2^-benzo[*d*][1,3,2]diazagermole (350 mg, 0.707 mmol) were dissolved in 10 mL of toluene, in a Schlenk with a Teflon stopcock, and heated at 110 °C for 18 h. The orange solution was transferred to a Schlenk and evaporated to dryness. The crude product was washed with pentane (3 × 2 mL), at −78 °C, to afford a pale yellow solid: yield 213 mg (48%). Crystals suitable for X-ray diffraction analysis were obtained from a concentrated solution of the solid in pentane at −30 °C in the glovebox. Anal. Calcd for C_25_H_52_GeN_2_OsP_2_: C, 42.56; H, 7.43; N, 3.97. Found: C, 42.84; H, 7.47; N, 4.22. HRMS (electrospray, *m*/*z*): calcd for C_25_H_49_GeN_2_OsP_2_ [M – 3H]^+^, 705.2192; found, 705.2213. IR (cm^–1^): ν(OsH) 2064 (w). ^1^H NMR (300 MHz, benzene-*d*_6_, 298 K): δ 7.18–7.02 (m, 2H, Ph), 6.98 (d, ^3^*J*_H–H_ = 7.6, 1H, Ph), 6.76 (d, ^3^*J*_H–H_ = 7.6, 1H, Ph), 3.24 (s, 3H, NCH_3_), 2.96 (d, ^2^*J*_H–P_*=* 4.1, 2H, NCH_2_), 1.72 (sept, ^3^*J*_H–H_ = 6.9, 3H, C*H*(CH_3_)_2_), 1.25 (d, ^3^*J*_H–P_ = 12.3, 18H, ^*t*^Bu), 1.18 (dd, ^3^*J*_H–P_ = 13.1, ^3^*J*_H–H_ = 6.9, 18H, PCH(C*H*_3_)_2_), −10.45 (dd, ^2^*J*_H–P_ = ^2^*J*_H’–P′_ = 12.4, 4H, OsH_4_). ^1^H NMR (400 MHz, toluene-*d*_*8*_, 193 K, high-field region): δ −10.40 (br dd, ^2^*J*_H–P_ = ^2^*J*_H′–P′_ = 12.8 Hz, 4 H, OsH_4_). *T*_1_(min) (ms, 400 MHz, toluene-*d*_8_, 223 K): 220 ± 22 (−10.45 ppm). ^31^P{^1^H} NMR (121 MHz, benzene-*d*_6_, 298 K): δ 99.8 (d, ^2^*J*_P–P_ = 224.5), 55.5 (d, ^2^*J*_P–P_ = 224.5). ^13^C{^1^H}-APT NMR plus HSQC and HMBC (75 MHz, benzene-*d*_6_, 298 K): δ 144.2 (s, C_q_-Ph), 140.3 (d, ^3^*J*_C–P_ = 12.1, C_q_-Ph), 119.0 (s, Ph), 117.5 (s, Ph), 110.0 (s, Ph), 108.3.0 (s, Ph), 35.1 (dd, ^1^*J*_C–P_ = 19.3, ^3^*J*_C–P_ = 1.7, C_q_-^*t*^*Bu*), 32.5 (s, NCH_3_), 31.8 (d, ^1^*J*_C–P_ = 25.5, NCH_2_P), 30.2 (d, ^2^*J*_C–P_ = 4.0, CH_3_-^*t*^Bu), 29.4 (dd, ^1^*J*_C–P_ = 24.5, ^3^*J*_C–P_ = 2.1, *C*H(CH_3_)_2_), 21.06 (s, CH(*C*H_3_)_2_).

### Reaction of **2** with Benzoic Acid

Compound **2** (100 mg, 0.142 mmol) and benzoic acid (19.0 mg, 0.156 mmol) in 5 mL of toluene were stirred for 16 h at room temperature. After evaporation to dryness, 3 mL of pentane was added to form a pale yellow solid of complex **3**, which was washed with pentane (3 × 2 mL): yield 66 mg (56%). Crystals suitable for an X-ray diffraction analysis were obtained from a concentrated solution of the solid in pentane at −30 °C in the glovebox. Anal. Calcd for C_32_H_56_GeN_2_O_2_OsP_2_: C, 46.55; H, 6.84; N, 3.39. Found: C, 46.31; H, 6.56; N, 3.78. HRMS (electrospray, *m*/*z*): calcd for C_32_H_54_GeN_2_O_2_OsP_2_ [M – 2H]^+^, 826.2482; found, 826.2478. IR (cm^–1^): ν(OsH) 1995 (m); ν(C–O) 1530 (s); 1488 (s). ^1^H NMR (300 MHz, toluene-*d*_*8*_, 298 K): δ 7.92 (dd, ^3^*J*_H–H_*=* 8.0, ^4^*J*_H–H_*=* 1.8, 2H, PhCOO), 7.16 (m, 1H, Ph), 7.10 (m, 1H, Ph), 7.03 (m, 3H, Ph), 6.85 (m, 2H, Ph), 3.53 (s, 3H, NCH_3_), 3.24 (dd, ^2^*J*_H–P_*=* 12.6, ^2^*J*_H–H_*=* 1.8, 1H, NCH_2_), 2.53 (dd, ^2^*J*_H–P_*=* 12.4, ^2^*J*_H–H_*=* 1.8, 1H, NCH_2_), 1.78 (m, 3H, PC*H*(CH_3_)_2_), 1.22 (dd, ^2^*J*_H–P_ = 13.0, ^3^*J*_H–H_ = 7.1, 9H, PCH(C*H*_3_)_2_), 1.06 (dd, ^2^*J*_H–P_ = 13.0, ^3^*J*_H–H_ = 7.1, 9H, PCH(C*H*_3_)_2_), 1.12 (d, ^3^*J*_H–P_ = 12.8, 9H, P^*t*^Bu_2_), 1.01 (d, ^3^*J*_H–P_ = 11.9, 9H, P^*t*^Bu_2_), −11.55 (br, 3H, OsH_3_). ^1^H NMR (300 MHz, toluene-*d*_8_, 183 K, high-field region): δ −3.64 (br s, 1H, OsH), −15.12 (AB system, Δν = 1080 Hz, *J*_A–B_ = 162 Hz, 2 H, OsH_2_). *T*_1_(min) (ms, Os–H, 300 MHz, toluene-*d*_8_, 223 K): 64 ± 6 (−16.69 ppm); the *T*_1_(min) value of the resonance at −4.08 ppm could not be calculated due to its broadness. ^31^P{^1^H} NMR (121 MHz, toluene-*d*_8_, 298 K): δ 84.6 (d, ^2^*J*_P–P_ = 236.5), 49.2 (d, ^2^*J*_P–P_ = 236.5). ^13^C{^1^H}-APT NMR plus HSQC and HMBC (75 MHz, toluene-*d*_8_, 298 K): δ 176.2 (s, C_q_-COO), 145.3 (s, C_q_-Ph), 140.6 (d, ^3^*J*_C–P_ = 12.8, C_q_-Ph), 140.5 (s, C_q_-Ph), 132.1 (s, Ph), 130.5 (s, Ph), 128.3 (s, Ph), 117.5 (s, Ph), 116.5 (s, Ph), 108.5 (s, Ph), 107.4 (s, Ph), 37.1 (d, ^1^*J*_C–P_ = 22.4, C_q_-P^*t*^Bu_2_), 36.1 (dd, ^1^*J*_C–P_ = 18.2, ^3^*J*_C–P_ = 2.7, C_q_-P^*t*^Bu_2_), 32.8 (s, N*C*H_3_), 31.2 (d, ^1^*J*_C–P_ = 26.4, N*C*H_2_), 29.4 (dd, ^2^*J*_C–P_ = 19.3, ^4^*J*_C–P_ = 3.3, *C*H_3_-^*t*^Bu), 26.7 (dd, ^1^*J*_C–P_ = 23.6, ^3^*J*_C–P_ = 1.9, *C*H(CH_3_)_2_), 20.5 (s, CH(*C*H_3_)_2_), 20.0 (s, CH(*C*H_3_)_2_).

### Reaction of **2** with Acetic Acid

A solution of complex **2** (100 mg, 0.142 mmol) in 5 mL of toluene was treated with glacial acetic acid (9.4 mg, 0.156 mmol, 9 μL). The mixture was stirred for 4 h at room temperature. After evaporation of the solvent, the residue was extracted with pentane (3 × 1 mL). The liquors were evaporated to dryness to afford a pale yellow solid of complex **4**: yield 52 mg (48%). Anal. Calcd for C_27_H_54_GeN_2_O_2_OsP_2_: C, 42.47; H, 7.13; N, 3.67. Found: C, 42.73; H, 7.39; N, 4.04. HRMS (electrospray, *m*/*z*): calcd for C_27_H_52_GeN_2_O_2_OsP_2_ [M – 2H]^+^, 764.2325; found, 764.2314. IR (cm^–1^): ν(OsH) 2001 (m); ν(C–O) 1546 (s), 1488 (s). ^1^H NMR (300 MHz, benzene-*d*_6_, 298 K): δ 7.09 (m, 2H, Ph), 6.89 (m, 2H, Ph), 3.44 (s, 3H, NCH_3_), 3.26 (dd, ^2^*J*_H–P_*=* 12.7, ^2^*J*_H–H_*=* 1.5, 1H, NCH_2_), 2.62 (dd, ^2^*J*_H–P_*=* 12.3, ^2^*J*_H–H_*=* 1.5, 1H, NCH_2_), 1.81 (m, 6H, C*H*_3_COO + PC*H*(CH_3_)_2_), 1.25–0.95 (m, 36H, PCH(C*H*_3_)_2_ + P^*t*^Bu_2_), −11.65 (br, 3H, OsH_3_). ^1^H NMR (300 MHz, toluene-*d*_8_, 183 K, high-field region): δ −3.83 (br s, 1H, OsH), −15.37 (AB system, Δν = 1083 Hz, *J*_A–B_ = 124.5 Hz, 2 H, OsH_2_). *T*_1_(min) (ms, Os–H, 300 MHz, toluene-*d*_8_, 218 K): 41 ± 4 (−17.17 ppm); the *T*_1_(min) value of the resonance at −4.04 ppm could not be calculated due to the broadness of it. ^31^P{^1^H} NMR (121 MHz, benzene-*d*_6_, 298 K): δ 84.96 (d, ^2^*J*_P–P_ = 237.5), 48.32 (d, ^2^*J*_P–P_ = 237.5). ^13^C{^1^H}-APT NMR plus HSQC and HMBC (75 MHz, benzene-*d*_6_, 298 K): δ 181.6 (s, C_q_-COO), 145.5 (s, C_q_-Ph), 140.7 (d, ^3^*J*_C–P_ = 12.8, C_q_-Ph), 117.4 (s, Ph), 116.6 (s, Ph), 108.6 (s, Ph), 107.4 (s, Ph), 37.2 (d, ^1^*J*_C–P_ = 22.7, C_q_-P^*t*^Bu_2_), 36.3 (d, ^1^*J*_C–P_ = 17.7, C_q_-P^*t*^Bu_2_), 32.7 (s, N*C*H_3_), 31.4 (d, ^1^*J*_C–P_ = 26.5 Hz, NCH_2_), 29.6 (dd, ^2^*J*_C–P_ = 25.4, ^4^*J*_C–P_ = 3.4, *C*H_3_-^*t*^Bu), 27.0 (d, ^1^*J*_C–P_ = 23.4, *C*H(CH_3_)_2_), 20.3 (d, ^2^*J*_C–P_ = 17.1, CH(*C*H_3_)_2_), 19.8 (s, *C*H_3_COO).

### Reaction of **2** with Formic Acid: Characterization of **5**

Compound **2** (10 mg, 0.0142 mmol) and formic acid (0.7 mg, 0.0156 mmol, 0.6 μL) were dissolved in 0.5 mL of toluene-*d*_8_ in a Young-NMR tube. ^1^H NMR (300 MHz, toluene-*d*_8_, 298 K): δ 7.01 (m, 2H, Ph), 6.81 (m, 2H, Ph), 6.63 (s, 1H, HCOO), 3.43 (s, 3H, NCH_3_), 3.21 (dd, ^2^*J*_H–P_*=* 12.1, ^2^*J*_H–H_*=* 1.7, 1H, NCH_2_), 2.49 (dd, ^2^*J*_H–P_*=* 12.3, ^2^*J*_H–H_*=* 1.7, 1H, NCH_2_), 1.74 (m, 3H, PC*H*(CH_3_)_2_), 1.18 (dd, ^3^*J*_H–P_ = 12.6, ^3^*J*_H–H_ = 6.9, 9H, PCH(C*H*_3_)_2_), 1.12 (d, ^3^*J*_H–P_ = 12.7, 9H, P^*t*^Bu), 1.05 (dd, ^3^*J*_H–P_ = 12.8, ^3^*J*_H–H_ = 6.9, 9H, PCH(C*H*_3_)_2_), 0.96 (d, ^3^*J*_H–P_ = 12.1, 9H, P^*t*^Bu), −11.73 (br, 3H, OsH_3_). ^1^H NMR (300 MHz, toluene-*d*_8_, 183 K, high-field region): δ −4.16 (br s, 1H, OsH), −15.66 (AB system, Δν = 1175 Hz, *J*_A–B_ = 153.0 Hz, 2 H, OsH_2_). *T*_1_(min) (ms, Os–H, 300 MHz, toluene-*d*_8_, 223 K): 64 ± 6 (−17.28 ppm); the *T*_1_(min) value of the resonance at −4.32 ppm could not be calculated due to its broadness. ^31^P{^1^H} NMR (121 MHz, toluene-*d*_8_, 298 K): δ 84.1 (d, ^2^*J*_P–P_ = 234.6), 48.3 (d, ^2^*J*_P–P_ = 234.6). ^13^C{^1^H}-APT NMR plus HSQC and HMBC (75 MHz, toluene-*d*_8_, 298 K): δ 173.2 (s, H*C*OO), 145.5 (s, C_q_-Ph), 140.7 (d, ^3^*J*_C–P_ = 12.9, C_q_-Ph), 117.9 (s, Ph), 117.0 (s, Ph), 108.9 (s, Ph), 107.8 (s, Ph), 37.6 (d, ^1^*J*_C–P_ = 22.7, C_q_-^*t*^Bu), 36.8 (dd, ^1^*J*_C–P_ = 18.3, ^3^*J*_C–P_ = 2.8, C_q_-^*t*^Bu), 33.0 (s, N*C*H_3_), 31.4 (d, ^1^*J*_C–P_ = 26.5, NCH_2_), 29.8 (dd, ^3^*J*_C–P_ = 12.4, ^5^*J*_C–P_ = 3.3, CH_3_-^*t*^Bu), 27.0 (dd, ^1^*J*_C–P_ = 23.7, ^3^*J*_C–P_ = 1.9, *C*H(CH_3_)_2_), 20.4 (s, CH(*C*H_3_)_2_).

### General Procedure for the Formic Acid Dehydrogenation Studies

The progress of the reaction was monitored using a reactor equipped with a pressured transducer (Man on the Moon series X103 kit; https://www.manonthemoontech.com/x102-gas-evolution.html). The total volume of the reactor was 18.8 mL. The procedure employed was as follows: under an argon atmosphere, a solution of **2** in 1 mL of toluene was incorporated into the reactor, which was closed and placed in an oil bath at the desired temperature. Then, the pressure was monitored until a stable value was reached and the reactor tared. Subsequently the formic acid was injected through a septum cap. This moment was considered the initial time of the catalysis. The reaction was followed by measuring the total pressure as a function of the time.

### NMR Spectroscopic Studies of the Decarboxylation of Complex **5**

The decarboxylation of complex **5** was monitored by ^31^P{^1^H} NMR spectroscopy (162.0 MHz). NMR spectra were acquired in a temperature range from 318 to 338 K at different times. A 0.5 mL portion of a 0.0142 M solution of **2** in toluene-*d*_8_ was placed in a Young NMR tube with a capillary containing a solution of PPh_3_ as an internal standard. The addition of 10 μL of a 0.7 M solution of HCOOH afforded the instantaneous formation of **5**. The ^31^P{^1^H} NMR parameters were modified as follows for the integration of the signal: pulse program (zgig30), *d*_1_ ≥ 5*T*_1_ (*d*_1_ = 33 s). Prior to the study, the relaxation times of **2**, **5**, and PPh_3_ were determined by varying the delay times.

## Supporting Information Available

General information, structural analysis of complexes **2** and **5**, computational data, and NMR spectra (PDF)
Cartesian coordinates of computed complexes (XYZ)

## Supplementary Material

ic1c02893_si_001.pdf

ic1c02893_si_002.xyz

## Accession Codes

CCDC 2096535–2096536 contain the supplementary crystallographic data for this paper. These data can be obtained free of charge via www.ccdc.cam.ac.uk/data_request/cif, or by emailing data_request@ccdc.cam.ac.uk, or by contacting The Cambridge Crystallographic Data Centre, 12 Union Road, Cambridge CB2 1EZ, UK; fax: +44 1223 336033.
