# Supplementary material for: Alternative Conceptual Approach to the Design of Bifunctional Catalysts: An Osmium Germylene System for the Dehydrogenation of Formic Acid
Source: Inorg Chem. 2021 Oct 16;60(21):16860–70. doi: 10.1021/acs.inorgchem.1c02893 (PMC8564761; doi:10.1021/acs.inorgchem.1c02893)
Supplement: Supplementary file 1 — ic1c02893_si_001.pdf [file ic1c02893_si_001.pdf]

Supporting Information

Alternative Conceptual Approach to the Design of Bifunctional Catalysts: An Osmium-Germylene System for the Dehydrogenation of Formic Acid

María L. Buil,[a] Javier A. Cabeza,[b] Miguel A. Esteruelas,[,a] Susana Izquierdo,[a] Carlos J. Laglera-Gandara,[b] Antonio I. Nicasio,[a] Enrique Oñate [a]*

[a]Departamento de Química Inorgánica, Instituto de Síntesis Química y Catálisis Homogénea (ISQCH), Centro de Innovación en Química Avanzada (ORFEO-CINQA), Universidad de Zaragoza-CSIC, 50009 Zaragoza, Spain

[b]Departamento de Química Orgánica e Inorgánica, Centro de Innovación en Química Avanzada (ORFEO-CINQA), Universidad de Oviedo, 33071 Oviedo, Spain

*Corresponding author's e-mail address: M.A.E.: maester@unizar.es.

Contents:

Experimental Section: General Information **S3**

Structural Analysis of Complexes 2 and 3 **S3**

Theoretical Calculations **S4**

Energies of the optimized complexes **S5**

^{1}H NMR spectrum, ^{1}H NMR spectra as a function of the temperature, $^{31}P\{^{1}H\}$ NMR spectrum, and $^{13}C\{^{1}H\}$ spectrum of complex 2 (Figures S01 – S04) **S11**

^{1}H NMR spectrum, ^{1}H NMR spectra as a function of the temperature, $^{31}P\{^{1}H\}$ NMR spectrum, and $^{13}C\{^{1}H\}$ spectrum of complex 3 (Figures S05 – S08) **S13**

^{1}H NMR spectrum, ^{1}H NMR spectra as a function of the temperature, $^{31}P\{^{1}H\}$ NMR spectrum, and $^{13}C\{^{1}H\}$ spectrum of complex 4 (Figures S09– S12) **S15**

^{1}H NMR spectrum, ^{1}H NMR spectra as a function of the temperature, ^{31}P{^{1}H} NMR spectrum, and ^{13}C{^{1}H} spectrum of complex 5 (Figures S13 – S16) **S17**

References **S19**

Experimental Section: General Information

^{1}H, $^{31}P\{^{1}H\}$, and $^{13}C\{^{1}H\}$ NMR spectra were recorded on either a Bruker ARX 300 MHz, Bruker Avance 300 MHz or Bruker Avance 400 MHz instruments. C, H and N analyses were carried out in a PerkinElmer 2400 CHNS/O analyzer. High-resolution electrospray mass spectra (HRMS) were acquired using a MicroTOF-Q hybrid quadrupole time-of-flight spectrometer (Bruker Daltonics, Bremen, Germany).

Structural Analysis of Complexes 2 and 3.

X-ray data were collected for the complex on a Bruker APEX CCD diffractometer equipped with a normal or fine focus, and 2.4 kW sealed tube source (Mo radiation, λ = 0.71073 Å). Data were collected over the complete sphere covering 0.3° in ω. Data were corrected for absorption by using a multiscan method applied with the SADABS program.[1] The structures were solved by Patterson or direct methods and refined by full-matrix least squares on F^2 with SHELXL2016,[2] including isotropic and subsequently anisotropic displacement parameters. The hydrogen atoms were observed in the last Fourier Maps or calculated, and refined freely or using a restricted riding model. The hydride ligands were observed in the difference Fourier maps and refined with a restrained distance to osmium atoms (d_{Os-H} = 1.59 Å).

Crystal data for **2**: $C_{25}H_{52}GeN_2OsP_2$, M_W 705.41, yellow, irregular block (0.205 x 0.113 x 0.093 mm^3), monoclinic, space group $P2_1/n$, *a*: 8.2963(5) Å, *b*: 15.7391(9) Å, *c*: 22.1713(12) Å, *β*: 97.9460(10)°, *V* = 2867.2(3) Å^3, *Z* = 4, *Z'* = 1, D_{calc}: 1.634 g cm^{-3}, F(000): 1416, T = 100(2) K, μ 5.602 mm^{-1}. 45985 measured reflections (2θ: 3-57°, ω scans 0.3°), 7046 unique (R_{int} = 0.0349); min./max. transm. Factors 0.638/0.862. Final agreement factors were R^1 = 0.0201 (6187 observed reflections, $I > 2\sigma(I)$) and wR^2 =

0.0464; data/restraints/parameters 7046/3/ 305; GoF = 1.019. Largest peak and hole 1.306 (close to osmiun atoms) and -0.546e/ Å^3.

Crystal data for **3**: $C_{32}H_{56}GeN_2O_2OsP_2$, M_W 825.51, colourless, irregular block (0.210 x 0.150 x 0.104 mm^3), orthorhombic, space group Pca2_1, *a*: 17.5682(11) Å, *b*: 12.2657(8) Å, *c*: 15.7715(10) Å, V = 3398.5(4) Å^3, Z = 4, Z' = 1, D_{calc}: 1.613 g cm^{-3}, F(000): 1664, T = 100(2) K, μ 4.744 mm^{-1}. 33302 measured reflections (2θ: 3-57º, ω scans 0.3º), 8245 unique (R_{int} = 0.0447); min./max. transm. Factors 0.636/0.862. Final agreement factors were R^1 = 0.0294 (7256 observed reflections, I > 2σ(I)) and wR^2 = 0.0628; Flack parameter: -0.017(8); data/restraints/parameters 8245/10/ 378; GoF = 1.020. Largest peak and hole 1.036 (close to osmiun atoms) and -0.724 e/ Å^3.

Theoretical Calculations.

DFT calculations were carried out using the wB97XD functional[3], which includes the second generation of Grimme's dispersion interaction correction[4] as well as long-range interaction effects. This functional reproduces the local coordination geometry of transition-metal compounds very well, and it also corrects the systematic overestimation of nonbonded distances seen for all the density functionals that do not include estimates of dispersion.[5] The Stuttgart−Dresden relativistic effective core potential and the associated basis sets (SDD) were used for the Os[6] atoms. The basis set used for the remaining atoms was cc-pVDZ.[7] The SMD continuum model was used to model the effects of the solvent (toluene). All calculations were carried out with the Gaussian09 package.[8]

Geometries were fully optimized in vacuum (**2** and **3**) or in solution (mechanistic studies) without any geometry or symmetry constraints. Reactants, intermediates, and

products were characterized by frequency calculations,[9] and have positive definite Hessian matrices. Transition states were identified by having one imaginary frequency in the Hessian matrix. It was confirmed that transition states connect with the corresponding intermediates by means of application of an eigenvector corresponding to the imaginary frequency and subsequent optimization of the resulting structures. Gibbs energies were computed at 298.15 K and 1 atmosphere. All values collected in schemes and figures correspond to Gibbs energies in toluene in kcal mol^{-1}.

The coordinates for the computed structures can be found in the Supplementary Data File xyz.

Energies of the optimized complexes

2 (xray-vacuum)

Zero-point correction= 0.730908 (Hartree/Particle)
Thermal correction to Energy= 0.771128
Thermal correction to Enthalpy= 0.772072
Thermal correction to Gibbs Free Energy= 0.663222
Sum of electronic and zero-point Energies= -3943.133471
Sum of electronic and thermal Energies= -3943.093251
Sum of electronic and thermal Enthalpies= -3943.092306
Sum of electronic and thermal Free Energies= -3943.201157

3 (xray-vacuum)

Zero-point correction= 0.830272 (Hartree/Particle)
Thermal correction to Energy= 0.878091
Thermal correction to Enthalpy= 0.879035
Thermal correction to Gibbs Free Energy= 0.752715
Sum of electronic and zero-point Energies= -4362.613028
Sum of electronic and thermal Energies= -4362.565210
Sum of electronic and thermal Enthalpies= -4362.564266
Sum of electronic and thermal Free Energies= -4362.690585

5 (tol)

Zero-point correction= 0.747497 (Hartree/Particle)
Thermal correction to Energy= 0.790840
Thermal correction to Enthalpy= 0.791784
Thermal correction to Gibbs Free Energy= 0.676693
Sum of electronic and zero-point Energies= -4131.710987
Sum of electronic and thermal Energies= -4131.667645
Sum of electronic and thermal Enthalpies= -4131.666701
Sum of electronic and thermal Free Energies= -4131.781792

TS(5-6t$_a$) (tol)

Zero-point correction= 0.747817 (Hartree/Particle)
Thermal correction to Energy= 0.790656
Thermal correction to Enthalpy= 0.791600
Thermal correction to Gibbs Free Energy= 0.677557
Sum of electronic and zero-point Energies= -4131.677246
Sum of electronic and thermal Energies= -4131.634408
Sum of electronic and thermal Enthalpies= -4131.633463
Sum of electronic and thermal Free Energies= -4131.747506

6t$_a$ (tol)

Zero-point correction= 0.748184 (Hartree/Particle)
Thermal correction to Energy= 0.791978
Thermal correction to Enthalpy= 0.792922
Thermal correction to Gibbs Free Energy= 0.675626
Sum of electronic and zero-point Energies= -4131.680508
Sum of electronic and thermal Energies= -4131.636715
Sum of electronic and thermal Enthalpies= -4131.635771
Sum of electronic and thermal Free Energies= -4131.753067

TS(6ta-7ta) (tol)

Zero-point correction= 0.746293 (Hartree/Particle)
Thermal correction to Energy= 0.790014
Thermal correction to Enthalpy= 0.790958
Thermal correction to Gibbs Free Energy= 0.673296
Sum of electronic and zero-point Energies= -4131.672703
Sum of electronic and thermal Energies= -4131.628983
Sum of electronic and thermal Enthalpies= -4131.628039
Sum of electronic and thermal Free Energies= -4131.745700

7t$_a$ (tol)

Zero-point correction= 0.745394 (Hartree/Particle)
Thermal correction to Energy= 0.789971
Thermal correction to Enthalpy= 0.790916
Thermal correction to Gibbs Free Energy= 0.671265
Sum of electronic and zero-point Energies= -4131.679791
Sum of electronic and thermal Energies= -4131.635213
Sum of electronic and thermal Enthalpies= -4131.634269
Sum of electronic and thermal Free Energies= -4131.753920

TS(7t$_a$-8t) (tol)

Zero-point correction= 0.741137 (Hartree/Particle)
Thermal correction to Energy= 0.784885
Thermal correction to Enthalpy= 0.785829
Thermal correction to Gibbs Free Energy= 0.669129
Sum of electronic and zero-point Energies= -4131.649233
Sum of electronic and thermal Energies= -4131.605485
Sum of electronic and thermal Enthalpies= -4131.604541
Sum of electronic and thermal Free Energies= -4131.721241

8t (tol)

Zero-point correction= 0.741644 (Hartree/Particle)
Thermal correction to Energy= 0.786087
Thermal correction to Enthalpy= 0.787031
Thermal correction to Gibbs Free Energy= 0.668555
Sum of electronic and zero-point Energies= -4131.656270
Sum of electronic and thermal Energies= -4131.611827
Sum of electronic and thermal Enthalpies= -4131.610883
Sum of electronic and thermal Free Energies= -4131.729360

9t (tol)

Zero-point correction= 0.728076 (Hartree/Particle)
Thermal correction to Energy= 0.769018
Thermal correction to Enthalpy= 0.769963
Thermal correction to Gibbs Free Energy= 0.659109
Sum of electronic and zero-point Energies= -3943.123009
Sum of electronic and thermal Energies= -3943.082067
Sum of electronic and thermal Enthalpies= -3943.081123

Sum of electronic and thermal Free Energies= -3943.191977

TS(9t-2) (tol)

Zero-point correction= 0.725987 (Hartree/Particle)
Thermal correction to Energy= 0.766892
Thermal correction to Enthalpy= 0.767836
Thermal correction to Gibbs Free Energy= 0.655447
Sum of electronic and zero-point Energies= -3943.119450
Sum of electronic and thermal Energies= -3943.078545
Sum of electronic and thermal Enthalpies= -3943.077601
Sum of electronic and thermal Free Energies= -3943.189990

2 (tol)

Zero-point correction= 0.728628 (Hartree/Particle)
Thermal correction to Energy= 0.769577
Thermal correction to Enthalpy= 0.770522
Thermal correction to Gibbs Free Energy= 0.658830
Sum of electronic and zero-point Energies= -3943.156819
Sum of electronic and thermal Energies= -3943.115870
Sum of electronic and thermal Enthalpies= -3943.114925
Sum of electronic and thermal Free Energies= -3943.226617

TS(5-6t_b) (tol)

Zero-point correction= 0.744896 (Hartree/Particle)
Thermal correction to Energy= 0.788354
Thermal correction to Enthalpy= 0.789298
Thermal correction to Gibbs Free Energy= 0.672838
Sum of electronic and zero-point Energies= -4131.670006
Sum of electronic and thermal Energies= -4131.626548
Sum of electronic and thermal Enthalpies= -4131.625604
Sum of electronic and thermal Free Energies= -4131.742063

6t$_b$ (tol)

Zero-point correction= 0.744480 (Hartree/Particle)
Thermal correction to Energy= 0.789176
Thermal correction to Enthalpy= 0.790120
Thermal correction to Gibbs Free Energy= 0.670040
Sum of electronic and zero-point Energies= -4131.675200
Sum of electronic and thermal Energies= -4131.630503
Sum of electronic and thermal Enthalpies= -4131.629559
Sum of electronic and thermal Free Energies= -4131.749639

TS(6t$_b$-7t$_b$) (tol)

Zero-point correction= 0.744528 (Hartree/Particle)
Thermal correction to Energy= 0.788228
Thermal correction to Enthalpy= 0.789172
Thermal correction to Gibbs Free Energy= 0.672669
Sum of electronic and zero-point Energies= -4131.673171
Sum of electronic and thermal Energies= -4131.629470
Sum of electronic and thermal Enthalpies= -4131.628526
Sum of electronic and thermal Free Energies= -4131.745029

7t$_b$ (tol)

Zero-point correction= 0.745169 (Hartree/Particle)
Thermal correction to Energy= 0.789143
Thermal correction to Enthalpy= 0.790087
Thermal correction to Gibbs Free Energy= 0.672709
Sum of electronic and zero-point Energies= -4131.677504
Sum of electronic and thermal Energies= -4131.633530
Sum of electronic and thermal Enthalpies= -4131.632586
Sum of electronic and thermal Free Energies= -4131.749964

TS(7t$_b$-2) (tol)

Zero-point correction= 0.740948 (Hartree/Particle)
Thermal correction to Energy= 0.784732
Thermal correction to Enthalpy= 0.785676
Thermal correction to Gibbs Free Energy= 0.668936
Sum of electronic and zero-point Energies= -4131.673631
Sum of electronic and thermal Energies= -4131.629847
Sum of electronic and thermal Enthalpies= -4131.628902
Sum of electronic and thermal Free Energies= -4131.745643

CO_2 (tol)

Zero-point correction= 0.011822 (Hartree/Particle)
Thermal correction to Energy= 0.014446
Thermal correction to Enthalpy= 0.015390
Thermal correction to Gibbs Free Energy= -0.008876
Sum of electronic and zero-point Energies= -188.525828
Sum of electronic and thermal Energies= -188.523204
Sum of electronic and thermal Enthalpies= -188.522260
Sum of electronic and thermal Free Energies= -188.546526

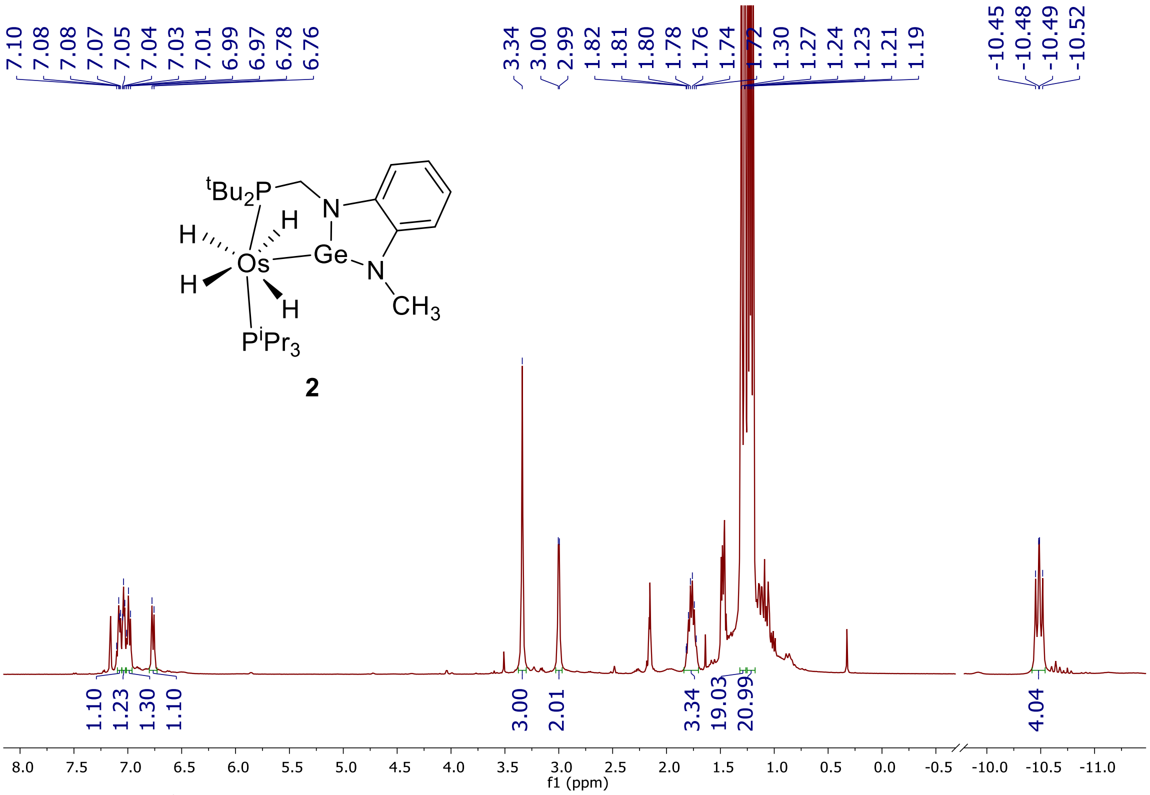

Figure S01. ^{1}H-NMR (300 MHz, toluene-d_8, 298 K) of complex **2**.

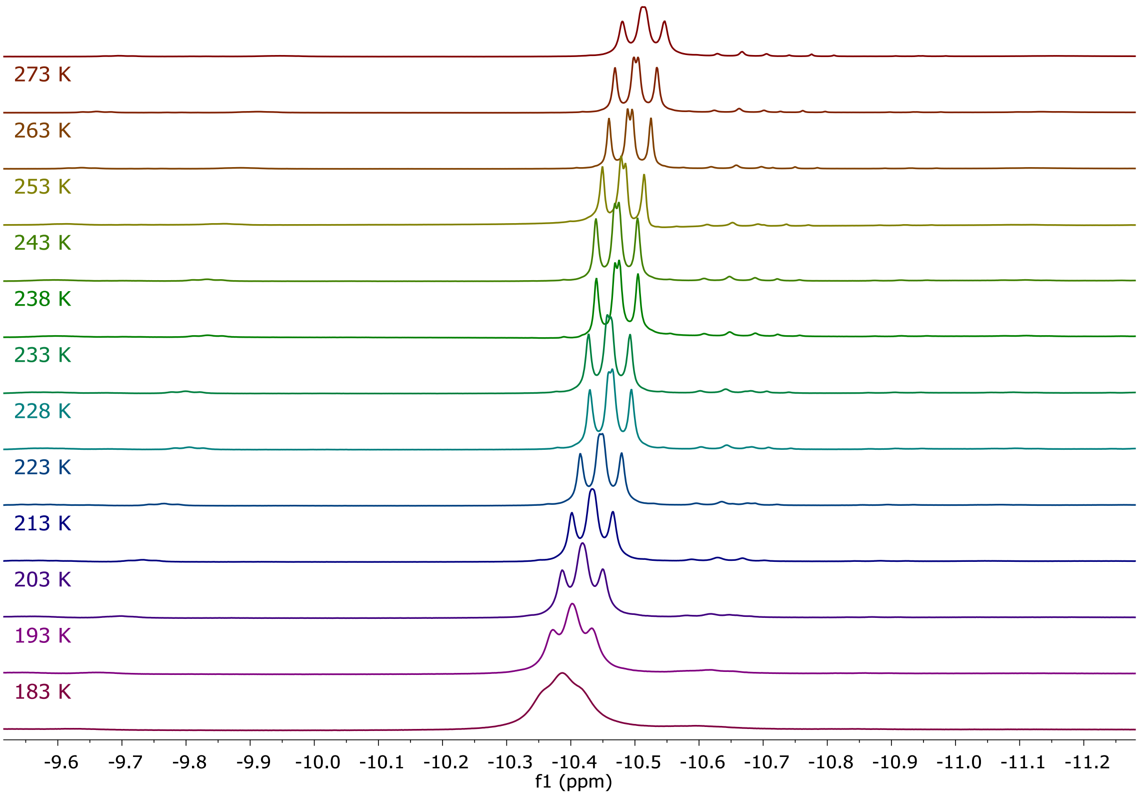

Figure S02. ^{1}H NMR as a function of the temperature (300 MHz, toluene-d_8) of complex **2**.

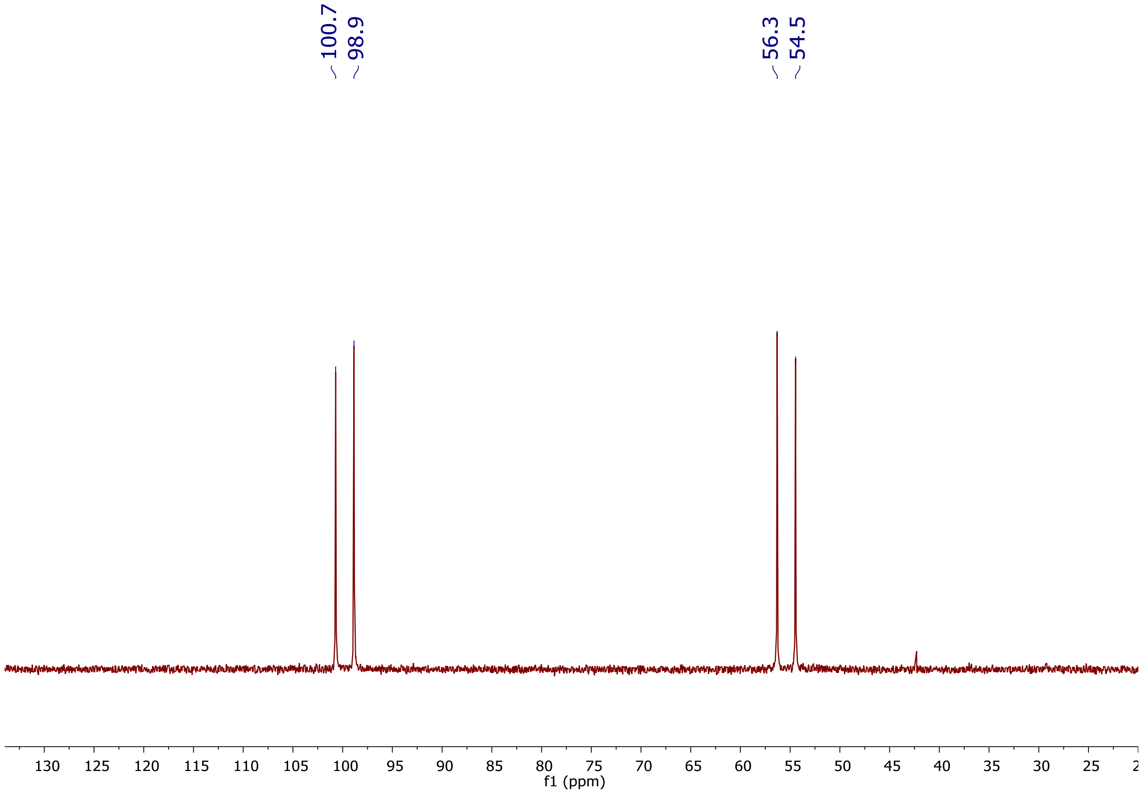

Figure S03. $^{31}P\{^1H\}$ NMR (121 MHz, benzene-d_6, 298 K) of complex **2**.

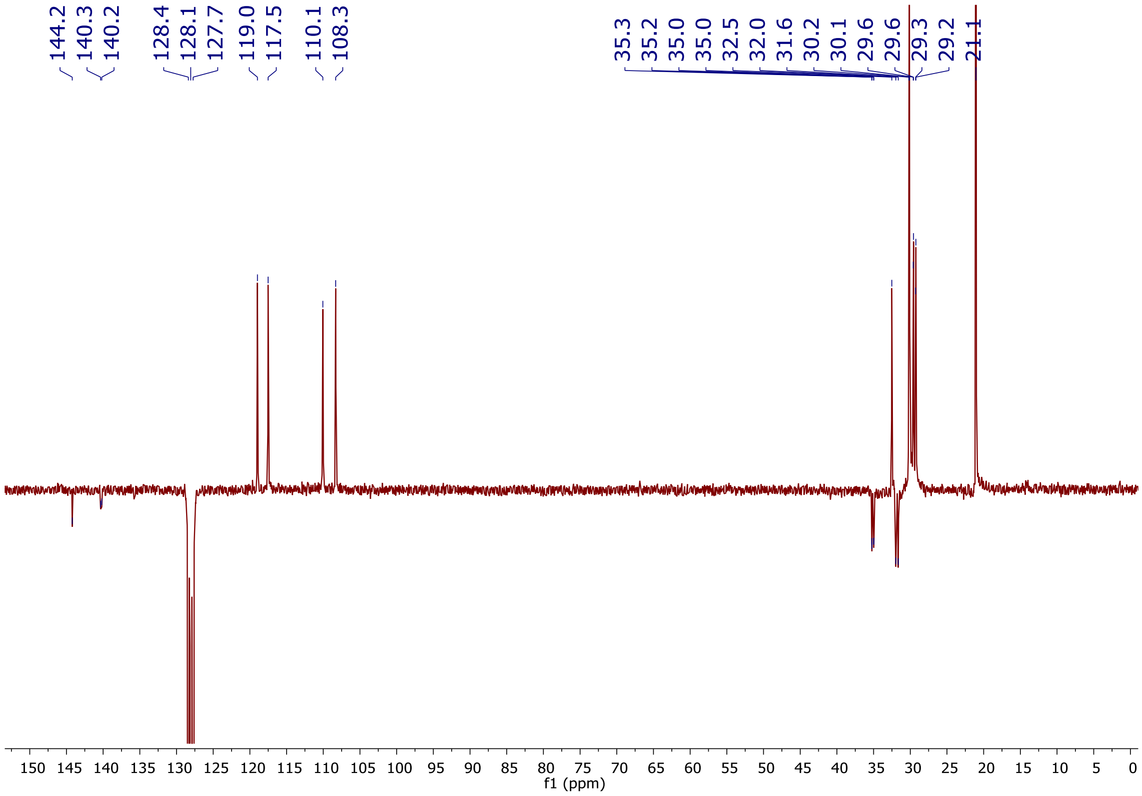

Figure S04. $^{13}C\{^1H\}$-APT NMR (75 MHz, benzene-d_6, 298 K) of complex **2**.

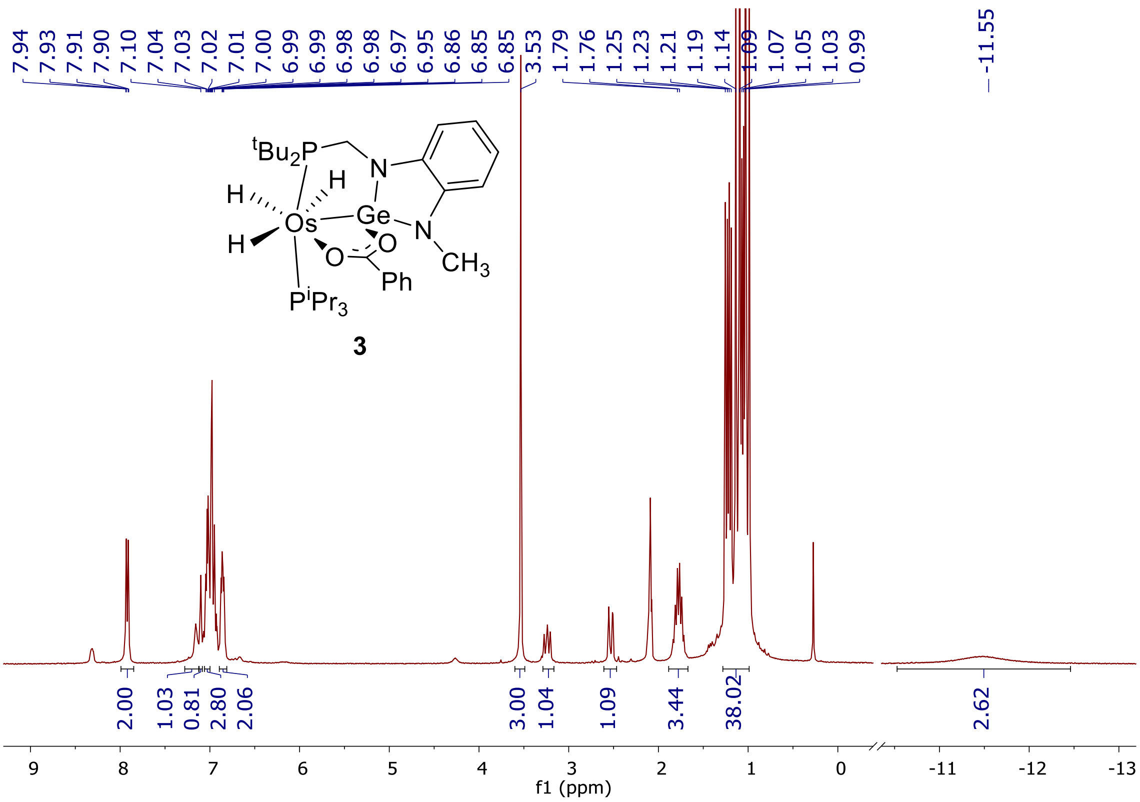

Figure S05. ^{1}H-NMR (300 MHz, toluene-d_8, 298 K) of complex **3**.

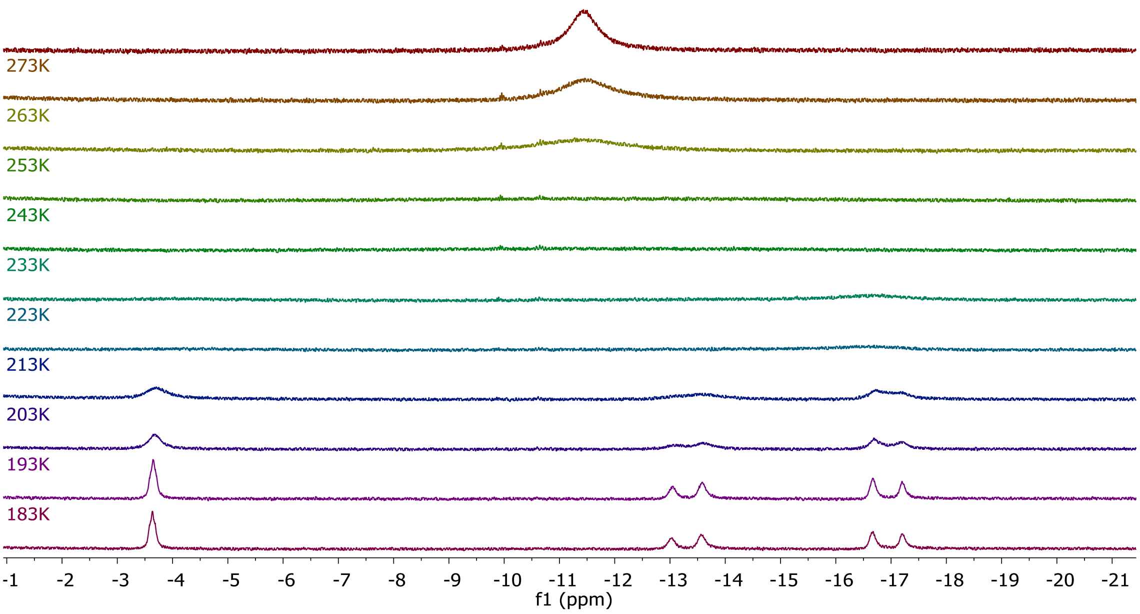

Figure S06. ^{1}H NMR as a function of the temperature (300 MHz, toluene-d_8) of complex **3**.

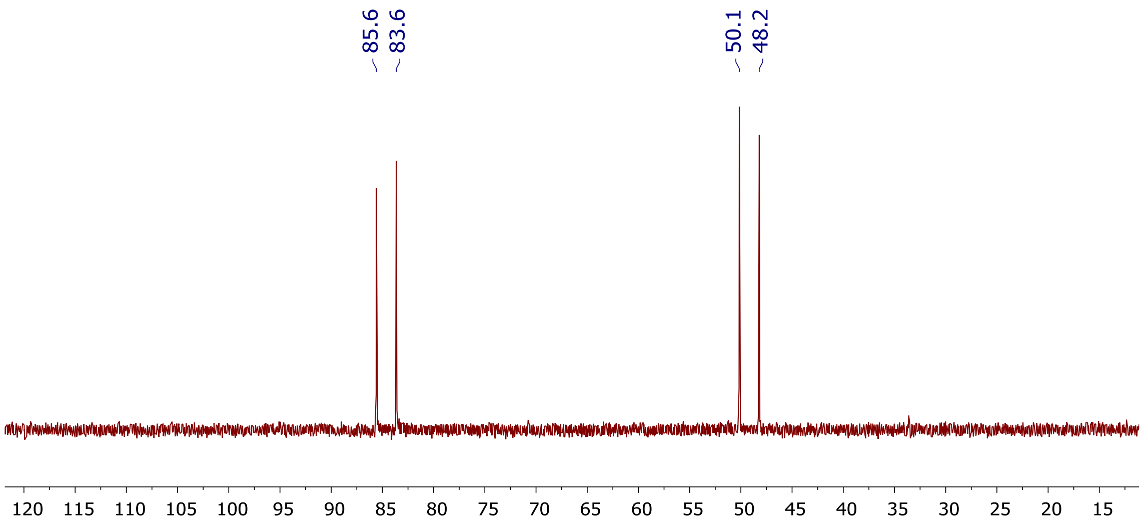

Figure S07. $^{31}P\{^{1}H\}$ NMR (121 MHz, toluene-d_8, 298 K) of complex **3**.

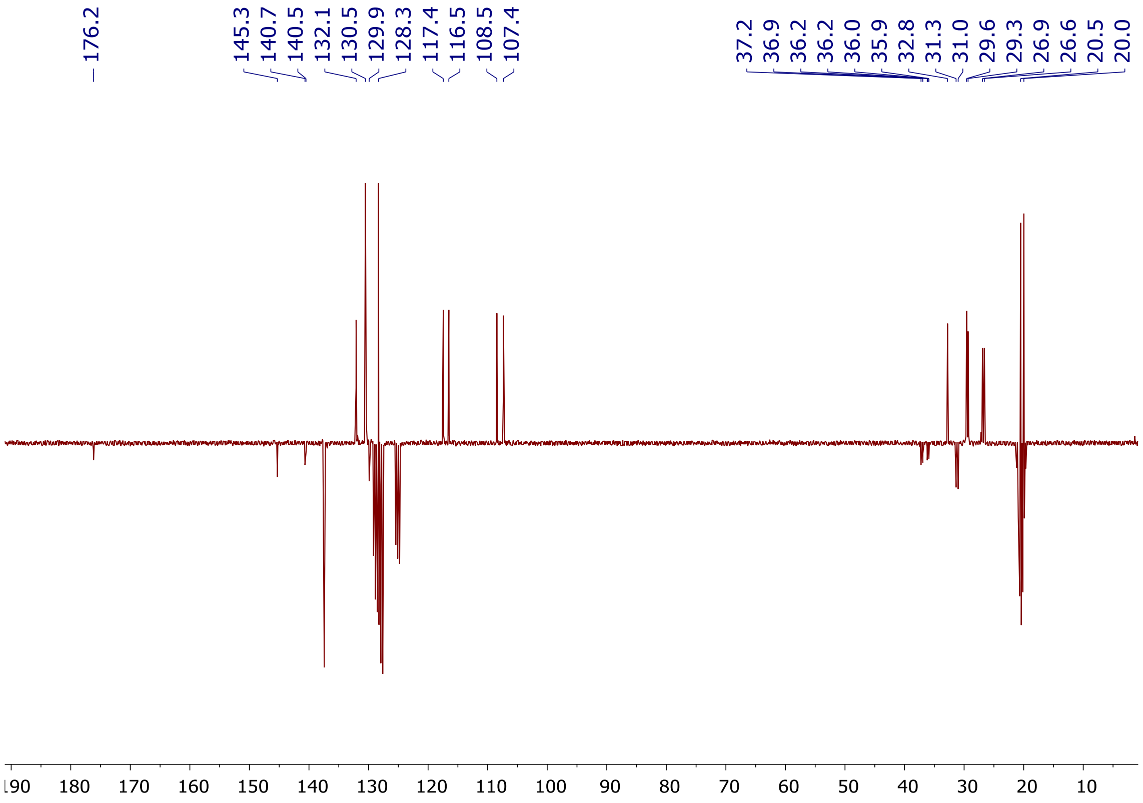

Figure S08. $^{13}C\{^{1}H\}$-APT NMR (75 MHz, toluene-d_8, 298 K) of complex **3**.

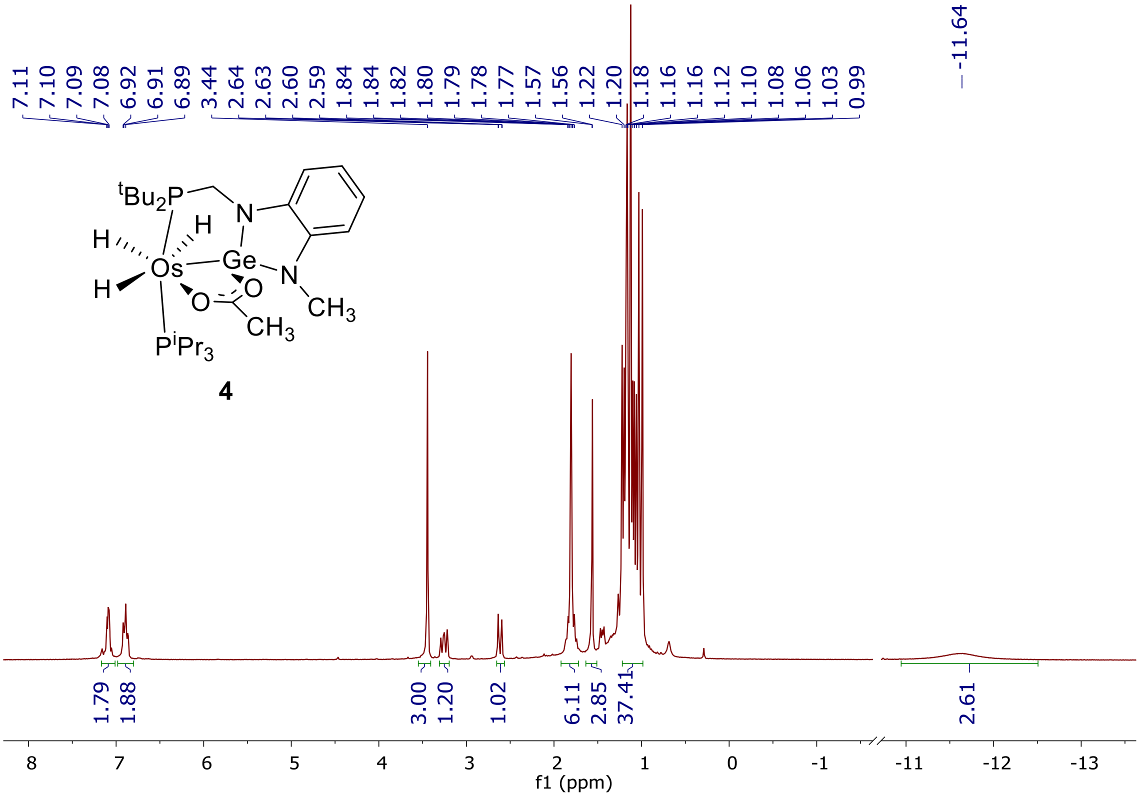

Figure S09. ^{1}H-NMR (300 MHz, benzene-d_6, 298 K) of complex **4**.

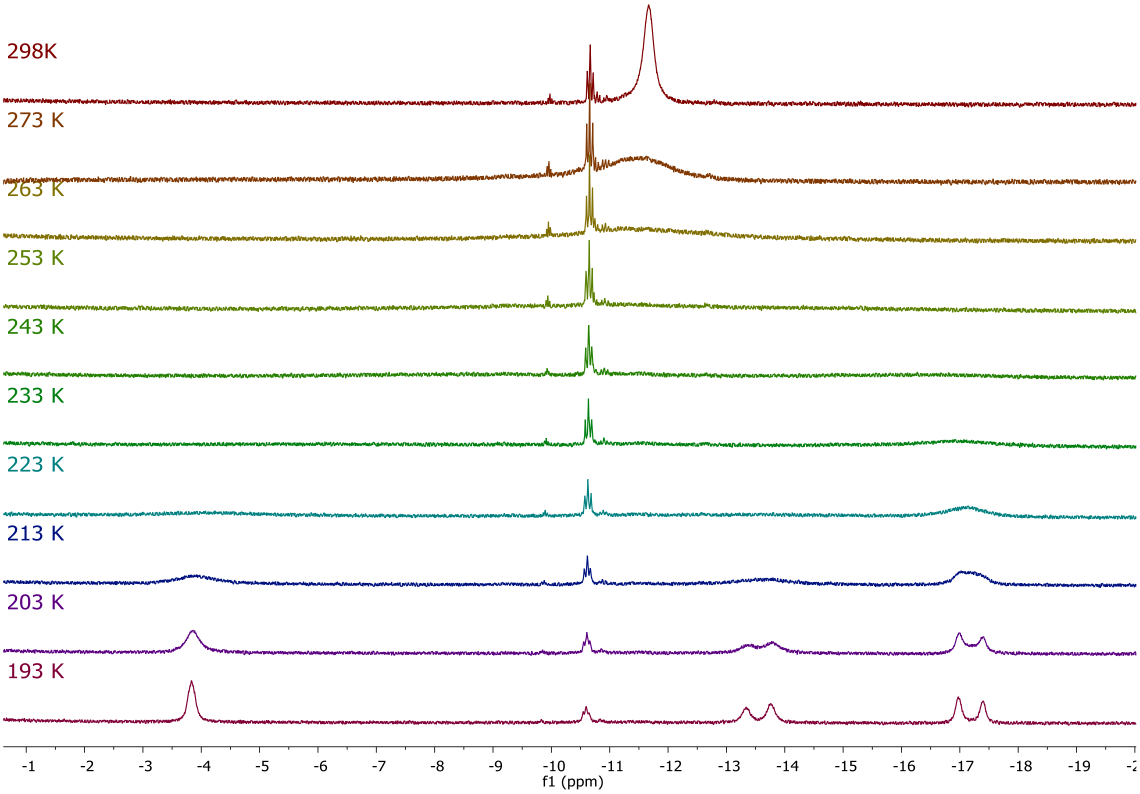

Figure S10. ^{1}H NMR as a function of the temperature (300 MHz, toluene-d_8) of complex **4**.

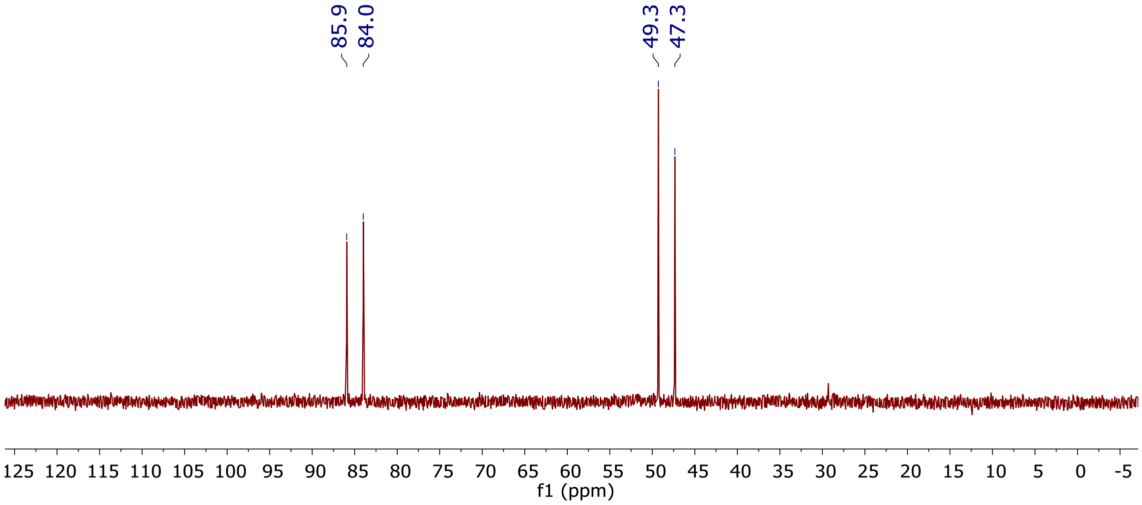

Figure S11. $^{31}P\{^{1}H\}$ NMR (121 MHz, benzene-d_6, 298 K) of complex **4**.

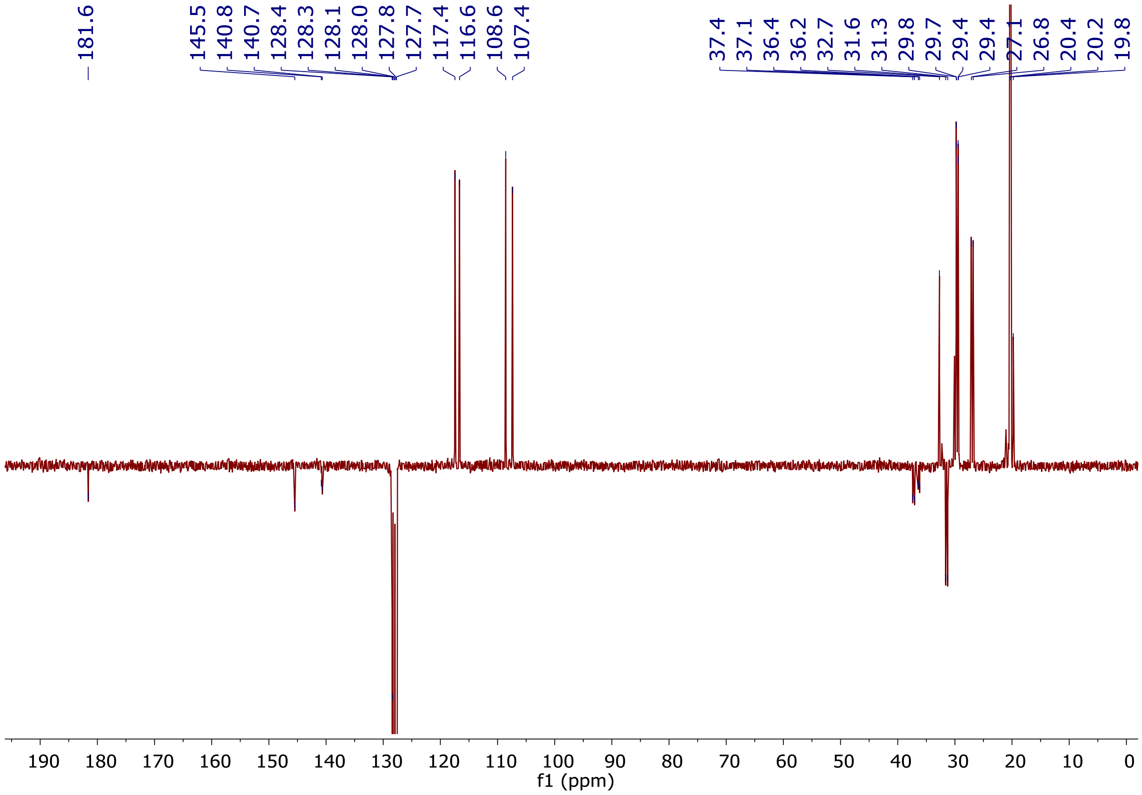

Figure S12. $^{13}C\{^{1}H\}$-APT NMR (75 MHz, benzene-d_6, 298 K) of complex **4**.

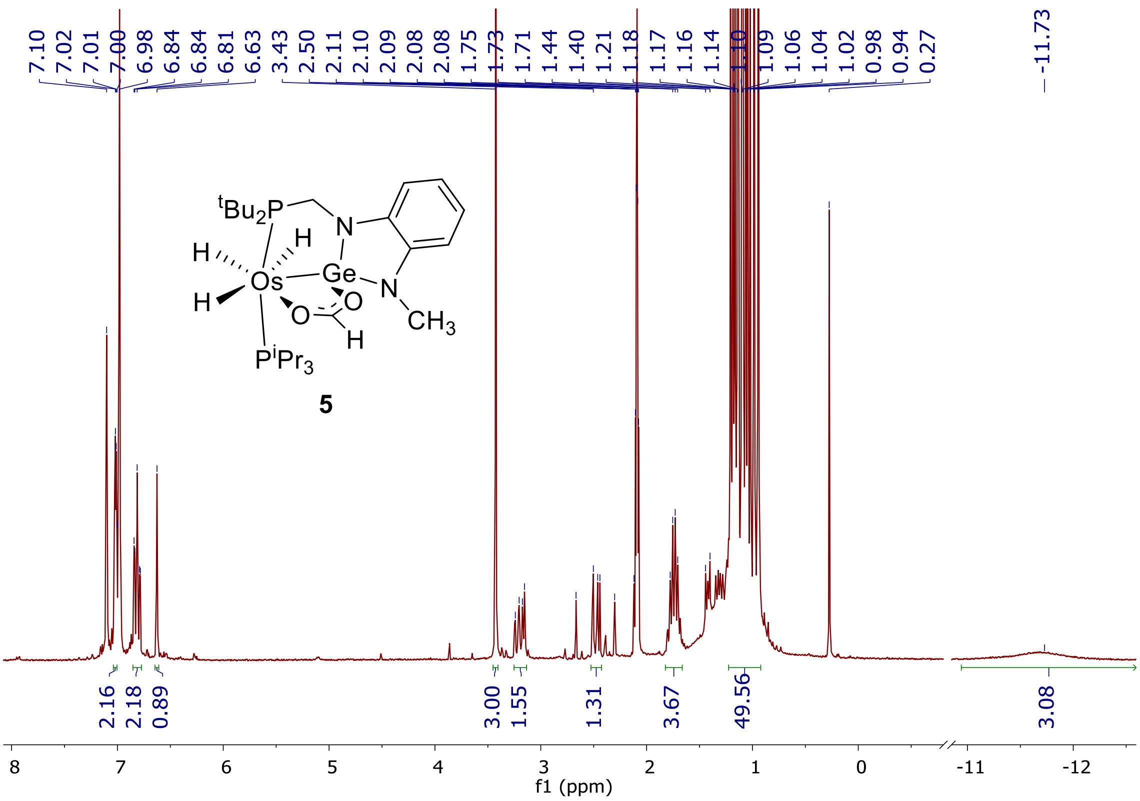

Figure S13. ^{1}H-NMR (300 MHz, benzene-d_6, 298 K) of complex **5**.

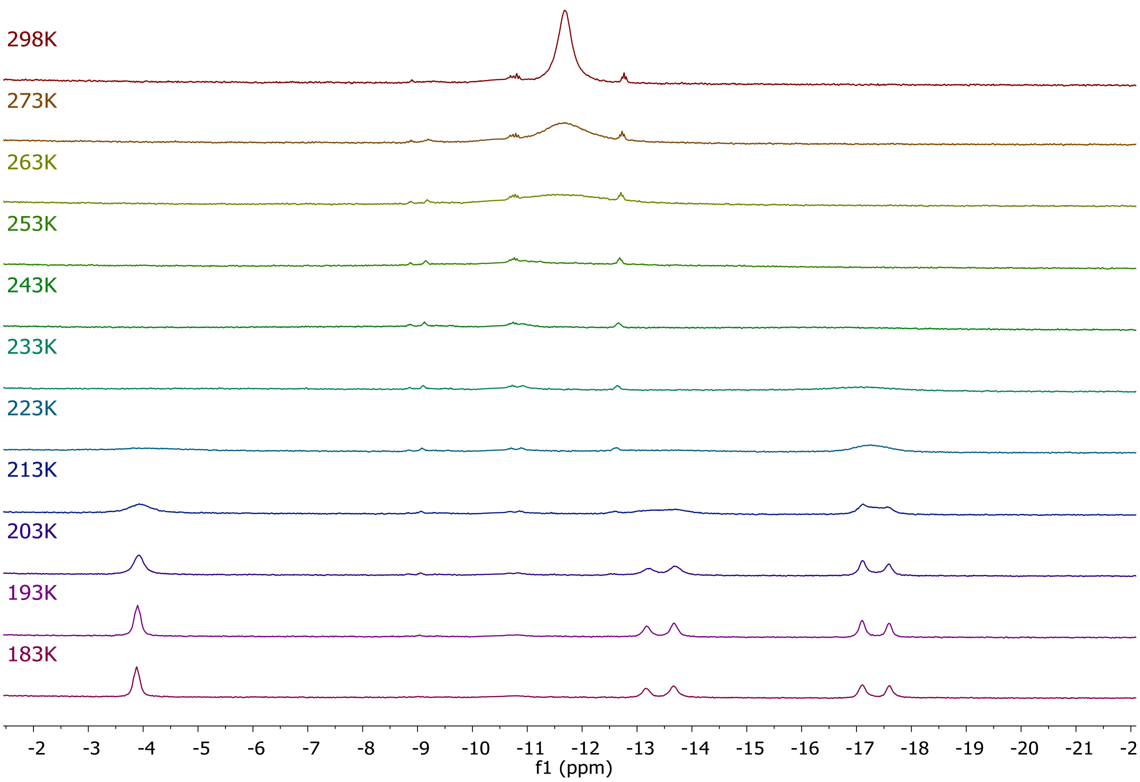

Figure S14. ^{1}H NMR as a function of the temperature (300 MHz, toluene-d_8) of complex **5**.

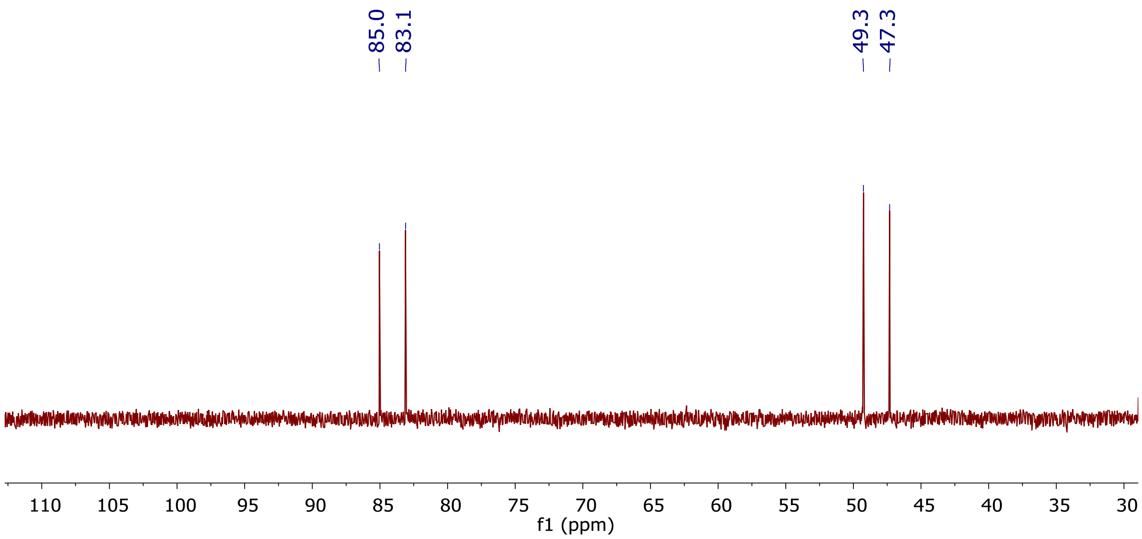

Figure S15. $^{31}P\{^{1}H\}$ NMR (121 MHz, benzene-d_6, 298 K) of complex **5**.

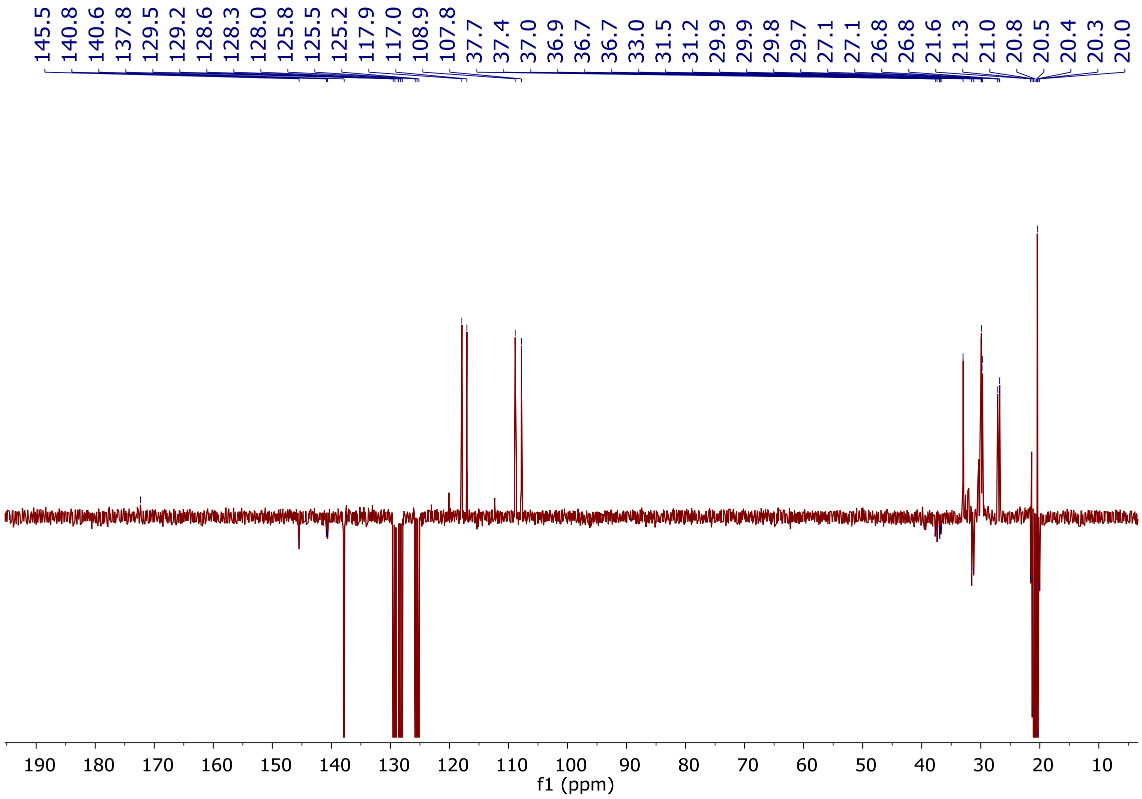

Figure S16. $^{13}C\{^{1}H\}$-APT NMR (75 MHz, toluene-d_8, 298 K) of complex **5**.

References

(1) Blessing, R. H. *Acta Crystallogr.* **1995**, *A51*, 33. SADABS: Area-detector absorption correction; Bruker- AXS, Madison, WI, 1996.

(2) SHELXL-2016/6. Sheldrick, G. M. *Acta Cryst.* **2008**, *A64*, 112-122.

(3) Chai, J.-D.; Head-Gordon, M. *Phys. Chem. Chem. Phys.* **2008**, *10*, 6615−6620.

(4) (a) Ehrlich, S.; Moellmann, J.; Grimme, S. *Acc. Chem. Res.* **2013**, *46*, 916−926. (b) Grimme, S. *Comp. Mol. Sci.* **2011**, *1*, 211−218. (c) Schwabe, T.; Grimme, S. *Acc. Chem. Res.* **2008**, *41*, 569−579.

(5) Minenkov, Y.; Singstad, Å.; Occhipinti, G.; Jensen, V. R. *Dalton Trans.* **2012**, *41*, 5526−5541.

(6) Figgen, D.; Peterson, K. A.; Dolg, M.; Stoll, H. *J. Chem. Phys.* **2009**, *130*, 164108−164120.

(7) Dunning, T. H. *J. Chem. Phys.* **1989**, *90*, 1007−1023.

(8) Gaussian 09, Revision E.01; Frisch, M. J.; Trucks, G. W.; Schlegel, H. B.; Scuseria, G. E.; Robb, M. A.; Cheeseman, J. R.; Scalmani, G.; Barone, V.; Mennucci, B.; Petersson, G. A.; Nakatsuji, H.; Caricato, M.; Li, X.; Hratchian, H. P.; Izmaylov, A. F.; Bloino, J.; Zheng, G.; Sonnenberg, J. L.; Hada, M.; Ehara, M.; Toyota, K.; Fukuda, R.; Hasegawa, J.; Ishida, M.; Nakajima, T.; Honda, Y.; Kitao, O.; Nakai, H.; Vreven, T.; Montgomery, J. A. Jr.; Peralta, J. E.; Ogliaro, F.; Bearpark, M.; Heyd, J. J.; Brothers, E.; Kudin, K. N.; Staroverov, V. N.; Kobayashi, R.; Normand, J.; Raghavachari, K.; Rendell, A.; Burant, J. C.; Iyengar, S. S.; Tomasi, J.; Cossi, M.; Rega, N.; Millam, J. M.; Klene, M.; Knox, J. E.; Cross, J. B.; Bakken, V.; Adamo, C.; Jaramillo, J.; Gomperts, R.; Stratmann, R. E.; Yazyev, O.; Austin, A. J.; Cammi, R.; Pomelli, C.; Ochterski, J. W.; Martin, R. L.; Morokuma, K.; Zakrzewski, V. G.; Voth, G. A.; Salvador, P.; Dannenberg, J. J.;

Dapprich, S.; Daniels, A. D.; Farkas, Ö.; Foresman, J. B.; Ortiz, J. V.; Cioslowski, J.; Fox, D. J.; Gaussian, Inc., Wallingford CT, 2013.

(9) McIver, J. W.; Komornicki, A. K. *J. Am. Chem. Soc.* **1972**, *94*, 2625-2633.
